# Olaparib-Resistant *BRCA2*^MUT^ Ovarian Cancer Cells with Restored BRCA2 Abrogate Olaparib-Induced DNA Damage and G2/M Arrest Controlled by the ATR/CHK1 Pathway for Survival

**DOI:** 10.3390/cells12071038

**Published:** 2023-03-29

**Authors:** Łukasz Biegała, Arkadiusz Gajek, Agnieszka Marczak, Aneta Rogalska

**Affiliations:** Department of Medical Biophysics, Institute of Biophysics, Faculty of Biology and Environmental Protection, University of Lodz, 90-236 Lodz, Poland

**Keywords:** ovarian cancer, targeted therapy, olaparib, PARP1, ATR/CHK1 pathway

## Abstract

The PARP inhibitor (PARPi) olaparib is currently the drug of choice for serous ovarian cancer (OC), especially in patients with homologous recombination (HR) repair deficiency associated with deleterious *BRCA1/2* mutations. Unfortunately, OC patients who fail to respond to PARPi or relapse after treatment have limited therapeutic options. To elucidate olaparib resistance and enhance the efficacy of olaparib, intracellular factors exploited by OC cells to achieve decreased sensitivity to PARPi were examined. An olaparib-resistant OC cell line, PEO1-OR, was established from *BRCA2*^MUT^ PEO1 cells. The anticancer activity and action of olaparib combined with inhibitors of the ATR/CHK1 pathway (ceralasertib as ATRi, MK-8776 as CHK1i) in olaparib-sensitive and -resistant OC cell lines were evaluated. Whole-exome sequencing revealed that PEO1-OR cells acquire resistance through subclonal enrichment of *BRCA2* secondary mutations that restore functional full-length protein. Moreover, PEO1-OR cells upregulate HR repair-promoting factors (BRCA1, BRCA2, RAD51) and PARP1. Olaparib-inducible activation of the ATR/CHK1 pathway and G2/M arrest is abrogated in olaparib-resistant cells. Drug sensitivity assays revealed that PEO1-OR cells are less sensitive to ATRi and CHK1i agents. Combined treatment is less effective in olaparib-resistant cells considering inhibition of metabolic activity, colony formation, survival, accumulation of DNA double-strand breaks, and chromosomal aberrations. However, synergistic antitumor activity between compounds is achievable in PEO1-OR cells. Collectively, olaparib-resistant cells display co-existing HR repair-related mechanisms that confer resistance to olaparib, which may be effectively utilized to resensitize them to PARPi via combination therapy. Importantly, the addition of ATR/CHK1 pathway inhibitors to olaparib has the potential to overcome acquired resistance to PARPi.

## 1. Introduction

Olaparib is the first-in-class approved poly(ADP-ribose) polymerase (PARP) inhibitor (PARPi) which is currently indicated as a monotherapy for the maintenance treatment of recurrent platinum-sensitive ovarian cancer (OC), regardless of *BRCA1/2* status [1]. However, treatment for relapsed epithelial OC (EOC) after PARPi is mainly limited to platinum-based chemotherapy, which achieves modest clinical efficacy [2]. Preliminary data from the OReO trial (NCT03106987) evaluating the clinical benefits of olaparib maintenance retreatment for EOC patients who relapse after initial PARPi indicate that secondary therapy with olaparib could improve progression-free survival irrespective of *BRCA1/2* status. However, overall survival has not been significantly improved in olaparib groups compared to placebo groups. The modest efficacy of re-challenge olaparib therapy has also been documented in a single-institution study [3].

About ~50% of high-grade serous OC (HGSOC) cases have defective homologous recombination (HR) repair, mainly due to *BRCA1* or *BRCA2* deleterious mutations. The remaining HR deficiency (HRD)-positive patients are defined as non-*BRCA1/2*^MUT^ due to harboring other biomarkers of HRD [4,5]. A recent ARIEL4 study demonstrated that intrinsic resistance to PARPi is associated with *BRCA1/2* reversion mutations in PARPi-naïve relapsed HGSOC patients previously treated with chemotherapy [6]. Indeed, an effective response to PARPi is typically dependent on defects emerging in the HR repair pathway due to the phenomenon of PARPi-induced synthetic lethality in tumors [7]. Interestingly, a proportion of HR-deficient OC patients are not responsive to olaparib therapy, indicating that current HRD assays are inadequate for the effective prediction of response to olaparib [8,9].

In addition to the suppression of PARP signaling, olaparib exerts so-called “PARP poisoning” cytotoxicity by trapping the PARP1 enzyme at DNA single-strand breaks, which eventually leads to the accumulation of double-strand breaks (DSBs) and contributes to its anticancer activity [10]. However, the therapeutic efficacy of olaparib is abolished in some cases because of intrinsic or acquired resistance of OC cells resulting from the restoration of HR proficiency (*BRCA1/2* reversion mutation, BRCA1 promoter demethylation), increased RAD51 foci formation, reduced PARP1 trapping, restoration of replication fork stabilization, and PARP signaling [11,12].

Numerous novel combination strategies with PARPi have been proposed to overcome resistance to olaparib [11,13,14], including small-molecule agents targeting the ATR (ataxia telangiectasia-mutated and Rad3-related) and CHK1 (checkpoint kinase 1) kinases implicated in the DNA damage response (DDR) and cell cycle regulation [15,16,17,18,19]. Concurrent treatments with PARPi and ATR or CHK1 kinase inhibitors have achieved promising results in patients who progress following initial therapy with PARPi, and these strategies are under further investigation [20,21,22]. However, approved combined therapy for the treatment of OC resistant to olaparib is lacking at present. The main rationale for combining PARPi with inhibitors of the ATR/CHK1 kinase axis is based on the observation that olaparib increases the reliance of olaparib-sensitive OC cells on the ATR/CHK1 pathway for survival [17,23]. DDR, which is regulated by the ATR/CHK1 axis, is a complex network of pathways integrating DNA replication and repair, cell cycle progression, and apoptosis to maintain genomic stability following replication stress and DNA damage [24,25]. However, the majority of HGSOC patients display loss of p53 and *TP53*^MUT^ tumor cells predominantly relying on S and G2/M cell cycle checkpoints strictly regulated by the ATR/CHK1 pathway. ATR, one of the most upstream DDR kinases that induce the signal transduction cascade following exposure to various DNA damage stimuli, is auto-phosphorylated at Thr1989. Following activation, ATR phosphorylates downstream effector proteins, including CHK1 kinase at Ser345 and histone H2AX at Ser139, eventually triggering DSB-induced checkpoint arrest and DNA repair mainly via error-free HR [26,27,28]. Indeed, the HR pathway is activated during the late S and G2 phases of the cell cycle and coordinated via ATR/CHK1-dependent control of S and G2/M checkpoints. In response, RAD51-dependent repair is promoted by BRCA1 and BRCA2 [28,29], the latter serving as the main mediator of RAD51 loading onto resected DSBs [28]. Alternatively, error-prone non-homologous end joining (NHEJ) regulated by 53BP1 may be utilized for DNA damage repair. However, this pathway is less accurate and potentially leads to chromosomal instability [4].

Resensitization of OC cells to PARPi is a key measure in expanding the therapeutic utility of olaparib. In the current study, we successfully generated an olaparib-resistant cell line (PEO1-OR) from the parent HGSOC cell line, PEO1, and investigated the complex mechanisms mediating the phenotype of resistance to olaparib in OC in vitro. Subsequently, the differences in the involvement of the ATR/CHK1 pathway in response to PARPi in olaparib-sensitive and -resistant HGSOC cell lines were determined, and the effects of concurrent treatment with olaparib and ATR/CHK1 blockage on DDR and cell cycle progression were examined.

## 2. Materials and Methods

### 2.1. Chemicals

PARPi (O, olaparib, AZD2281), ATRi (A, ceralasertib, AZD6738), and CHK1i (C, MK-8776) were purchased from Selleck Chemicals (Houston, TX, USA). The stock solutions of studied inhibitors were separately prepared from powders dissolved in 100% dimethyl sulfoxide (DMSO), aliquoted, and stored at −80 °C for up to a maximum of six months. RPMI 1640 and DMEM culture media, heat-inactivated fetal bovine serum (HI-FBS), and trypsin-EDTA were obtained from Gibco (Thermo Fisher Scientific, Waltham, MA, USA). Details concerning other key reagents used in the studies are included in Section 2 and Supplementary Tables (Tables S1–S3). Chemicals and solvents were obtained from Sigma-Aldrich (Saint Louis, MO, USA) or Avantor Performance Materials Poland S.A. (Gliwice, Poland).

### 2.2. Cell Lines

Human HGSOC PEO1 and PEO4 cell lines were purchased from ECACC and upon receipt prepared for cryopreservation following recovery of frozen stocks and culture expansion. HGSOC cell lines were cultured as monolayers in RPMI 1640 culture medium containing GlutaMAX supplement and HEPES and supplemented with 10% HI-FBS. Human hepatocellular carcinoma HepG2 cell line was purchased from ATCC and cultured as monolayers in high-glucose DMEM culture medium containing GlutaMAX supplement and HEPES without sodium pyruvate and supplemented with 10% HI-FBS. All cell lines were cultured at 37 °C in a humidified atmosphere containing 5% CO_2_.

### 2.3. Development of Olaparib Resistance in HGSOC Cell Line In Vitro

The human HGSOC cell line resistant to olaparib (PEO1-OR) was derived from a parental PEO1 cell line in our laboratory by constant exposure to gradually increasing concentrations of olaparib by 2-fold (10–80 μM) during culture of cells growing in log phase in RPMI 1640 culture medium containing 10% HI-FBS over the period of 20 days. Primarily used doses of olaparib were based on an IC_50_ value of olaparib determined from a dose–response curve over 5 days in the parental cell line. The preliminarily acquired cell line was cultured in the medium deprived of olaparib for additional 72 days to adapt cells to PARPi-free conditions and maintain and ensure its increased stable resistance to the drug. The sensitivity of PEO1-OR cells to olaparib was re-assessed periodically by MTT cell viability assay throughout the entire process of development to estimate a fold-change of resistance with respect to the PEO1 cell line. Briefly, the cells were exposed to a range of doses of olaparib (0–120 μM or 0–240 μM), and calculated IC_50_ values were compared between the established and the parental cell line to define the increase in drug resistance. Immediately after the establishment of the cell line, PEO1-OR cells were expanded from the homogeneous cell suspension for 10 days, banked in RPMI 1640 medium (supplemented with 10% DMSO and 20% HI-FBS), and stored in the vapor phase of liquid nitrogen for future use to ensure genetic stability. Fresh cultures were initiated every 2–3 months and cultivated in RPMI 1640 culture medium containing 10% HI-FBS in the absence of olaparib to conduct biological assays.

### 2.4. Cell Proliferation Rate and Population Doubling Time

Population doubling times (PDTs) of in vitro cultured PEO1, PEO1-OR, and PEO4 cells were determined based on the cell growth curves. Briefly, cells were seeded in 6-well plates (4 × 10^5^ PEO1 and PEO1-OR cells or 3 × 10^5^ PEO4 cells) containing 2 mL of RPMI 1640 culture medium with 10% HI-FBS and cultured over a period of 9 days (37 °C, 5% CO_2_) until they reached confluency and started to overgrow which finally resulted in growth inhibition. The medium was changed on the fourth and seventh days to ensure suitable nutritional conditions for growth. The number of viable and non-viable cells was examined using the trypan blue exclusion method and a Thoma chamber under a light microscope. Each cell line was seeded in three technical replicates on each plate, and the number of cells was counted twice for each sample. The experiment was repeated three times (*n* = 3). Cellular growth curves were generated by plotting viable cell counts against the time of culture and using a four-parameter logistic nonlinear regression model that best fitted a set of experimental data. The following formula was used to calculate PDT for the cells exhibiting a logarithmic phase of growth based on the growth curves:

PDT = (t_2_ − t_1_)/log_2_(N_2_/N_1_)

where N_1_ and N_2_—number of viable cells at earlier and later timepoints of the logarithmic phase of growth, t_1_ and t_2_—timepoints for N_1_ and N_2_, respectively.

### 2.5. MTT Cell Viability Assay

Drug cytotoxic activity and its impact on cell viability, linked with cellular metabolic activity, was measured by MTT assay. Cells were seeded in 96-well plates (0.8 × 10^4^ PEO1 or PEO1-OR cells and 1.6 × 10^4^ PEO4 cells per well) in 100 μL of culture medium and incubated for 24 h (37 °C, 5% CO_2_). On the following day, 50 μL of fresh culture medium and 50 μL of 4 × concentrated working dilutions of drugs prepared in culture medium were added to each well. Then the cells were further incubated for two or five days (37 °C, 5% CO_2_). Following the treatment, the medium was aspirated, 50 μL of MTT solution (0.5 mg/mL in DPBS) was added to each well, and the plates were incubated for 4 h (37 °C, 5% CO_2_). Afterward, 100 μL of DMSO was added to each well, plates were incubated at room temperature (RT) or at 37 °C protected from light until complete solubilization of the formazan crystals, and the samples were mixed for about 30 s using a plate shaker. The absorbance was determined spectrophotometrically on a microplate reader (Synergy HTX, BioTek, Shoreline, WA, USA) at an experimental wavelength of 580 nm, using 720 nm as a reference wavelength. To determine cell survival, the absorbance at 720 nm was subtracted from the absorbance at 570 nm (A_580_ − A_720_) for individual wells. Relative cell viability was calculated as the percentage of untreated control cells using corrected absorbance values. A minimum of three independent experiments (*n* ≥ 3) with a minimum of three intraplate technical replicates were performed and the results were presented as mean ± standard deviation (SD). MTT assay was preliminarily used to plot dose–response curves for 5 days of olaparib (0.1–240 μM), ATRi, and CHK1i (0.1–120 μM) based on a four-parameter logistic model (generated by GraphPad Prism software) from which half-maximal (cytotoxic) inhibitory values (IC_50_) were calculated.

The total cytotoxic effect exhibited by concurrent administration of two inhibitors for five days was determined by the assessment of the coefficient of drug interaction (CDI) to select the lowest and most effective in olaparib-sensitive cell lines doses of olaparib (0.5, 2.5, 5, 10 μM) with either ATRi (0.5 μM) or CHK1i (2.5 μM) used in further experiments. The CDI was calculated according to the formula CDI = AB/(A × B) [30]. Based on the absorbance of each group, AB is the ratio of the two-agent combination group to the untreated control group, and A or B is the ratio of the single-agent group to the control group. CDI values allowed the characterization of whether the interaction effects were significantly synergistic (CDI < 0.7), synergistic (CDI < 1.0), additive (CDI = 1.0), or antagonistic (CDI > 1.0).

To examine the role of multidrug resistance (MDR) transporters in drug cytotoxicity and their influence on drug distribution, PEO1, PEO1-OR, and PEO4 cells were preincubated for 1 h in a culture medium containing the potent, non-competitive P-glycoprotein inhibitor tariquidar (0.1 μM; Sigma-Aldrich) followed by a co-incubation with pre-selected doses of tested compounds (5 μM olaparib, 0.5 μM ATRi, 2.5 μM CHK1i, or their combinations) for two days in the presence of tariquidar (0.1 μM). P-glycoprotein inhibitor verapamil was used as a positive control of inhibition of MDR1 in HepG2 cells. Briefly, HepG2 cells were preincubated for 1 h in culture medium containing verapamil (50 μM; Sigma-Aldrich) and subsequently co-incubated for 2 days with the MDR1 substrate doxorubicin (0.10, 0.25 and 0.50 μM; Sequoia Research Products Limited, Compton, UK) or tested compounds in the presence of verapamil (50 μM). Cell viability in the presence of MDR inhibitors (MDRi) was determined using MTT assay as described above.

### 2.6. Clonogenic Assay

The cytotoxic activity of drugs and its influence on cell survival, proliferation, and ability to form colonies were examined using the colony formation assay. Cells were seeded in 6-well plates in 1.5 mL of culture medium (2 × 10^3^ cells per well) and incubated for 24 h (37 °C, 5% CO_2_). The following day, cells were treated with pre-selected doses of tested compounds (5 μM olaparib, 0.5 μM ATRi, 2.5 μM CHK1i, or their combinations) by adding 100 μL of 16 × concentrated drugs prepared in culture medium and allowed to grow for five days in the presence of drugs. After five days, the medium was changed, and cells were allowed to grow and proliferate in drug-free medium for 10–14 days until single clones in control wells formed non-overlapping colonies. Then colonies were fixed with the mixture of methanol and glacial acetic acid (7:1 (*v*/*v*)) for 10 min, stained with 0.5% (*w*/*v*) crystal violet in 20% (*v*/*v*) ethanol for 20 min, thoroughly rinsed with deionized water to remove residual dye, and air-dried at RT. Each well was photographed, and fixed colonies consisting of at least 50 cells were counted manually. Surviving fractions (SFs) were calculated according to the following formula:

SFs = (number of colonies formed after treatment)/(number of colonies formed in a control well) × 100%

### 2.7. Cellular Morphology Changes

The morphology changes induced by tested compounds in PEO1, PEO1-OR, and PEO4 cells were evaluated by brightfield microscopy technique. Cells were seeded in 12-well plates (5 × 10^4^ cells) containing 1 mL of culture medium and incubated for 24 h (37 °C, 5% CO_2_). The following day 0.5 mL of fresh culture medium was added to each well and cells were treated for five days (37 °C, 5% CO_2_) with olaparib (5 μM), ATRi (0.5 μM), CHK1i (2.5 μM), or their combinations by adding 0.5 mL of 4 × concentrated working dilutions of drugs prepared in culture medium. Afterward, cellular morphology changes were imaged at 10× magnification using an inverted optical microscope (Olympus IX70, Tokyo, Japan).

### 2.8. Whole-Exome Sequencing and Variant Calling

Whole-exome sequencing (WES) was performed with the Agilent SureSelect Human All Exon V8 Kit (Agilent Technologies, Santa Clara, CA, USA) on PEO1 and PEO1-OR cell line tumor samples. Paired-end (2 × 150 bp) next-generation sequencing was performed on a NovaSeq 6000 Sequencing System (Illumina, San Diego, CA, USA) to obtain a mean coverage of more than 100×. Briefly, freshly thawed and recovered cells from passage 1 were seeded in 100 mm dishes (5 × 10^5^ cells in 8 mL of culture medium) and cultured for four days (37 °C, 5% CO_2_) until reaching 80% confluency. Afterward, cells were harvested by trypsinization, and genomic DNA was isolated and purified using GenElute Mammalian Genomic DNA Miniprep Kits (Sigma-Aldrich) according to the manufacturer’s instructions. The quality, integrity, and purity of isolated DNA were evaluated using agarose gel electrophoresis and a spectrophotometer. Genomic DNA was stored in an ultrapure, molecular biology grade Tris-HCl solution (10 mM, pH 8.0) at –80 °C for a week until downstream analysis.

Raw sequence data were aligned and annotated for individual samples to a reference sequence of the human genome (GRCh38). Duplicate reads were excluded from downstream analysis. Next, variants were called utilizing GATK’s Haplotype Caller and interpreted using Qiagen Clinical Insight Interpret Translational (QCI-IT) software. Briefly, SnpEff 4.3 prediction toolbox was used to annotate called variants and predict their effects on protein structure and function (gain of function, loss of function, normal function, or change of function). False-positive variants were filtered out using GATK’s Variant Filtration module.

Thereafter, we restricted our analysis to the interpretation of single-nucleotide variants (SNVs) and short insertions and deletions (indels) in genes encoding studied proteins, i.e., p53 (*TP53*, NM_000546.6), ATR (*ATR*, NM_001184.4), CHK1 (*CHEK1*, NM_001114121.2), PARP1 (*PARP1*, NM_001618.4), PARG (*PARG*, NM_003631.5), BRCA1 (*BRCA1*, NM_007294.4), BRCA2 (*BRCA2*, NM_000059.4), MDR1 (*ABCB1*, NM_001348945.2), 53BP1 (*TP53BP1*, NM_005657.4), H2AX (*H2AX*, NM_002105.3), and RAD51 (*RAD51*, NM_002875.5), using the somatic workflow of QCI-IT software. To improve the high confidence of detected variants, we kept high-quality calls that passed upstream pipeline filtering and all of the confidence filters (call quality ≥ 30, genotype quality ≥ 30, and read depth ≥ 10). To keep only rare variants, any variant with a maximum population frequency >5% in the gnomAD database was classified as benign and excluded from further interpretation (unless previously established as a pathogenic common variant), according to recommendations [31].

High-quality variants were classified based on an automatically computed evidence-based categorization considering their pathogenicity (pathogenic, likely pathogenic, uncertain significance, likely benign, benign) and actionability in terms of therapeutic, diagnostic, and prognostic clinical significance (tier I—strong, tier II—potential, tier III—unknown, tier IV—benign or likely benign) according to the guidelines [32,33].

### 2.9. Western Blotting

For analyses of protein expression levels, cells were seeded in 60 mm dishes (0.8 × 10^6^ cells) or 100 mm dishes (2.0 × 10^6^ cells) and incubated for 24 h (37 °C, 5% CO_2_) followed by treatment with pre-selected doses of tested compounds or their combinations for two days (5 μM olaparib, 0.5 μM ATRi, 2.5 μM CHK1i). Whole-cell lysates were prepared on ice by washing with ice-cold DPBS and scraping the cells in ice-cold RIPA buffer (25 mM Tris-HCl, 150 mM NaCl, 1% NP−40, 1% sodium deoxycholate, 0.1% SDS) supplemented with phenylmethylsulfonyl fluoride (1 mM PMSF), Halt Protease Inhibitor Cocktail (1 mM AEBSF, 800 nm aprotinin, 50 μM bestatin, 15 μM E64, 20 μM leupeptin, 10 μM pepstatin A, 5 mM EDTA), and Halt Phosphatase Inhibitor Cocktail (NaF, Na_3_VO_4_, Na_4_P_2_O_7,_ and β-glycerophosphate). Lysates were gathered in microcentrifuge tubes, sonicated with a probe sonicator (1 pulse, 10 s, 15% amplitude), centrifuged (14,000× *g*, 10 min, 4 °C), and transferred to new tubes. Samples were prepared in mPAGE 4X LDS Sample Buffer supplemented with 50 mM β-mercaptoethanol and heated for 10 min at 70 °C, and equal amounts of proteins (30 μg) were loaded into each lane. Then, proteins were separated by SDS-PAGE at 180–200 V for 30–45 min in mPAGE Bis-Tris gels in an electrophoresis tank (Mini-PROTEAN Tetra Cell, Bio-Rad, Hercules, CA, USA) using MOPS SDS running buffer and transferred using a semi-dry transfer system (Trans-Blot Turbo Blotting System, Bio-Rad) in mPAGE Transfer Buffer onto 0.45 μM PVDF membranes according to the manufacturer’s optimized instruction for mPAGE Bis-Tris gels. Membranes were blocked either with 5% non-fat milk in TBST for 1 h or with SuperBlock (TBS) Blocking Buffer for 15 min and washed with TBST (3 × 5 min). Analyzed proteins were immunoblotted with primary antibodies overnight at 4 °C followed by incubation with suitable secondary antibodies conjugated with horseradish peroxidase (HRP) for 1 h at RT. The chemiluminescence signal of immunoreactive proteins was obtained using enhanced chemiluminescent (ECL) substrates (SuperSignal West Pico PLUS or SuperSignal West Atto Ultimate for low-abundance proteins). Images were visualized and captured with Azure 300 Imaging System (Azure Biosystems, Dublin, CA, USA). Densitometric quantification of the bands was performed with ImageJ software (NIH, Bethesda, MD, USA). Each experiment for a quantitative analysis of protein expression was independently repeated at least three times (*n* ≥ 3).

The following antibodies were used for immunoblotting: anti-53BP1 (Merck, #MAB3802), anti-ATR (Thermo Fisher Scientific, #MA1-23158), anti-phospho-ATR (Thr1989) (CST, #30632), anti-BRCA1 (Cell Signaling Technology, #9010), anti-BRCA2 C-terminal (Thermo Fisher Scientific, #A300-005A), anti-BRCA2 N-terminal #1 (Thermo Fisher Scientific, #A303-434A), anti-BRCA2 N-terminal #2 (Thermo Fisher Scientific, # MA5-23942), anti-CHK1 (Thermo Fisher Scientific, #MA5-32180), anti-phospho-CHK1 (Ser345) (CST, #2348), anti-MDR1 (Thermo Fisher Scientific, #MA5-32282), anti-PARG (Thermo Fisher Scientific, MA5-27034), anti-PARP1 (CST, #9532), anti-RAD51 (Merck, #ABE257), anti-β-actin (Merck, #A1978), anti-γH2AX (Merck, #05-636). Antibody dilution factors and utilized dilution buffers are indicated in the Supplementary Information (Tables S2 and S3).

### 2.10. Immunofluorescence Analysis of γH2AX and RAD51 Foci Formation

Immunofluorescence microscopy was used to assess the co-localization of the surrogate DNA DSB biomarker γH2AX (a form of H2AX histone phosphorylated at Ser139) and DNA repair factor RAD51 in response to exposure to tested compounds or their combinations (5 μM olaparib, 0.5 μM ATRi, 2.5 μM CHK1i).

Cells were seeded in CELLview glass-bottom slides (1.2 × 10^4^ in 200 μL of culture medium per well) and incubated for 24 h (37 °C, 5% CO_2_). The following day, the medium was changed, and cells were exposed to pre-selected doses of tested compounds or their combinations for two days (5 μM olaparib, 0.5 μM ATRi, 2.5 μM CHK1i). Then cells were fixed with formaldehyde (4% (*w*/*v*) in DPBS 1X) for 15 min at RT, rinsed with DPBS 1X (3 × 5 min), blocked and permeabilized with 5% normal goat serum and 0.3% Triton X-100 in DPBS 1X for 1 h at RT, and co-stained for 2 h at RT with mouse anti-γH2AX (Sigma-Aldrich, #05-636) and rabbit anti-RAD51 (Sigma-Aldrich, #ABE257) monoclonal antibodies diluted with 1% BSA and 0.3% Triton X-100 in DPBS 1X. Next, the specimens were rinsed with DPBS 1X (3 × 5 min) and incubated for 1 h at RT with a mixture of two fluorochrome-conjugated secondary antibodies raised against primary antibodies from mouse (Goat Anti-mouse IgG (H + L) Highly Cross-Adsorbed Secondary Antibody, Alexa Fluor Plus 488, Thermo Fisher Scientific, #A32723) and rabbit (Goat Anti-rabbit IgG (H + L), F(ab’)_2_ Fragment, Alexa Fluor 555 Conjugate, CST, #4413) prepared in 1% BSA and 0.3% Triton X-100 in DPBS 1X. After rinsing with DPBS 1X, nuclei were stained by incubation with 300 nm DAPI for 3 min at RT, rinsed once with DPBS 1X, and mounted with DPBS 1X for imaging. Immunofluorescence images were acquired using an inverted confocal scanning microscope (SP-8, Leica Microsystems, Wetzlar, Germany) under 63× oil magnification.

### 2.11. Preparation of Metaphase Chromosomes for Structural Aberration Analysis

For analysis of chromosome instability identified as structural chromosomal aberrations induced by examined drugs, cells were seeded in 60 mm dishes (0.8 × 10^6^ cells), incubated for 24 h (37 °C, 5% CO_2_), and treated with tested compounds or their combinations for two days (5 μM olaparib, 0.5 μM ATRi, 2.5 μM CHK1i). Then the cells were incubated with colcemid (0.1 μg/mL) for 1 h (37 °C, 5% CO_2_) to arrest the cells at metaphase for chromosome analysis. Metaphase chromosomes were prepared for visualization based on the in-house modification of the procedure from Howe et al. [34]. Briefly, cells were washed with DPBS 1X, detached from the dishes by trypsinization, collected in tubes, and centrifuged (200× *g*, 10 min). The pellets were resuspended, and cells were incubated in a prewarmed to 37 °C hypotonic solution of 0.075 M KCl for 10 min at 37 °C in a water bath. Following centrifugation (200× *g*, 5 min), cells were fixed with a fixative solution (3:1 (*v*/*v*)), methanol and glacial acetic acid). After three cycles of subsequent centrifugation (200× *g*, 5 min), removal of supernatant, and addition of fresh fixative, fixed cells were resuspended in a fixative solution and added drop by drop onto microscope slides tilted at a 45° angle. Then the slides were dried out at RT for about 10 min and placed for 10 min in a Giemsa staining solution. Excessive amounts of dyes were rinsed off with distilled water. Metaphase chromosome spreads were visualized with a light microscope (Nikon Eclipse 50i) equipped with a DS-Fi3 camera (Nikon). Images were acquired at 100× magnification. From each biological replicate (*n* = 3), thirty-five metaphases were analyzed in terms of the presence of any structural chromosomal aberrations (chromosome and chromatid breaks, gaps, exchanges, ring chromosomes, pulverized chromosomes, dicentric chromosomes, or centromeric disruption) for a total of 105 metaphases scored for every sample.

### 2.12. Neutral Comet Assay

DNA damage in the form of DSBs was detected and measured at the level of individual cells using the neutral comet assay in response to exposure to studied inhibitors. Cells were seeded in 6-well plates (4 × 10^5^ cells) in 1.5 mL of culture medium, incubated for 24 h (37 °C, 5% CO_2_), and exposed to pre-selected doses of tested compounds or their combinations (5 μM olaparib, 0.5 μM ATRi, 2.5 μM CHK1i) for two days in fresh culture medium. Then cells were harvested by trypsinization, washed, resuspended in DPBS 1X to obtain single-cell suspensions, and counted. Duplicate samples containing 4000 cells in 0.75% low-melting-point agarose dissolved in DPBS 1X (pH 7.4) were spread on glass slides precoated with 0.5% normal-melting-point agarose and immersed in cooled lysis buffer (2.5 M NaCl, 100 mM EDTA, 10 mM Tris, 1% Triton X-100, pH 9.0) for 18–24 h at 4 °C. Subsequently, slides were equilibrated for 20 min in a running buffer (100 mM Tris, 300 mM sodium acetate, pH 9.0 adjusted with glacial acetic acid) and electrophoresed in a fresh running buffer for 60 min (0.41 V/cm, 50 mA). After DNA unwinding and separation, samples were rinsed in distilled water, stained with DAPI (2 μg/mL), and stored in a humidified chamber at 4 °C in darkness before further analysis. For each sample, at least fifty randomly selected comets were visualized at 200× magnification with an Eclipse fluorescence microscope (Nikon, Tokyo, Japan) attached to a COHU 4910 video camera (Cohu Inc., San Diego, CA, USA) equipped with a UV-1 filter block (359 nm excitation filter, and 461 nm barrier filter). Comet tails containing fragments of DSB-induced damaged DNA (% DNA in a tail relative to total DNA in a comet) were quantitatively measured using the Lucia-Comet v. 4.51 image analysis system (Laboratory Imaging, Prague, Czech Republic). Each comet assay was independently repeated three times (*n* = 3) with each sample repeated in two technical replicates during experiments to calculate the mean percentage of tail DNA positively correlating with DNA DSBs.

### 2.13. Cell Cycle Analysis with Quantification of γH2AX by Flow Cytometry

Cells were characterized in terms of their cell cycle position (G0/G1, S, and G2 + M phases) using the bromodeoxyuridine (BrdU) incorporation assay with BD Pharmingen FITC BrdU Flow Kit (BD Biosciences, San Jose, CA, USA) according to the manufacturer’s protocol. Simultaneously, phosphorylation of H2AX at Ser139 (γH2AX) was measured to detect signs of DNA DSB damage within specific cell cycle phases by multicolor flow cytometric analysis.

Briefly, cells were seeded in 60 mm dishes (0.8 × 10^6^ cells) in 3 mL of culture medium per well and allowed to attach for 24 h (37 °C, 5% CO_2_). The following day, the medium was changed, and cells were exposed to pre-selected doses of tested compounds or their combinations for two days (5 μM olaparib, 0.5 μM ATRi, 2.5 μM CHK1i). During the final 3 h of treatment, cells were pulsed with 10 μM BrdU. Cells were harvested by trypsinization, fixed, permeabilized, and co-stained for 30 min at RT with FITC-conjugated anti-BrdU antibody and PE-conjugated anti-γ-H2AX antibody (Thermo Fisher Scientific, #12-9865-42) following staining with 7-amino-actinomycin D (7-AAD) for total DNA content. Stained cells were immediately acquired by collecting 10,000 events using flow cytometry (LSR II, Becton Dickinson, San Jose, CA, USA).

Flow cytometric data were analyzed using FlowJo v.7.6.1 analyzing software (Ashland, OR, USA). Briefly, cell populations of interest were gated based on forward scatter (FSC) and side scatter (SSC) to exclude cellular debris. Then doublets were excluded from the analysis of single cells using FSC height vs. FSC area and SSC height vs. SSC area density plots. Finally, cellular events corresponding to suitable cell cycle phases were gated based on two-parameter density plots presenting the cellular content of BrdU (y axis) and 7-AAD (x axis). Gated cells were used for the quantitative analysis of the phosphorylation level of γH2AX using geometric mean fluorescence intensity (MFI) in arbitrary units in the entire gated population of interest (total γH2AX) and in populations from respective cell cycle phases (G0/G1, S, and G2/M).

### 2.14. Statistical Analysis

Statistical analysis was performed with GraphPad Prism version 9.4.0 for Windows (GraphPad Software, San Diego, CA, USA) using the tests specified in the description of the figures. One-way ANOVA followed by Tukey’s multiple comparison test was used to assess the statistical significance of differences in experiments where three independent groups defined by one factor (cell line) passed the assumption of normal distribution (Shapiro–Wilk or D’Agostino–Pearson test), and homogeneity of variance (Brown–Forsythe test). Welch’s ANOVA followed by Dunnett T3 multiple comparison test was applied to compare measurements from three independent groups defined by one factor (cell line) where applicable (data sampled from a Gaussian distribution, but with unequal variances across groups). Two-way ANOVA followed by Tukey’s multiple comparison test was used to assess the statistical significance of differences in experiments where three or more independent groups defined by two factors (cell line and drug treatment) passed the assumption of normal distribution (Shapiro–Wilk or D’Agostino–Pearson test).

## 3. Results

### 3.1. The PEO1-OR Cell Line with an Acquired Resistant Phenotype Displays Reduced Sensitivity to Olaparib and Increased Proliferation

**Figure 1:**
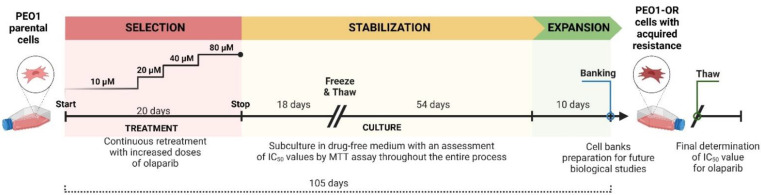
Timeline of a continuous selection strategy and stabilization for generating PEO1-OR cells with a phenotype of acquired resistance to olaparib. In the first round of selection, PEO1 parental cells were seeded at moderate confluence and exposed to 10 μM olaparib, and additional doses of drug were administered every 2–5 days during medium change or cell passage. Next, cells were sequentially cultured in the presence of stepwise increasing doses of olaparib (20, 40, and 80 μM) at each round of selection. After 20 days of treatment, cells were cultured in the absence of drug for a total of 72 days to stabilize changes in sensitivity to the drug and subjected to a single cycle of freeze–thaw to ensure the presence of the resistant phenotype. Eventually, the culture of selected cells was expanded and cryopreserved for future use. Untreated parental cells were cultured alongside those used for olaparib selection throughout the procedure.

PEO1-OR cells with acquired resistance to olaparib were established from the parental *BRCA2*^MUT^ PEO1 cell line to uncover the mechanisms underlying PARPi cytotoxicity and resistance in OC cells in vitro. A multiple-dose selection strategy was employed to gradually increase resistance in the presence of the drug (Figure 1). To ensure consistency in rationale-based repeated oral dosing of olaparib in clinical practice, OC cells were constantly exposed to olaparib for 20 days.

**Figure 2:**
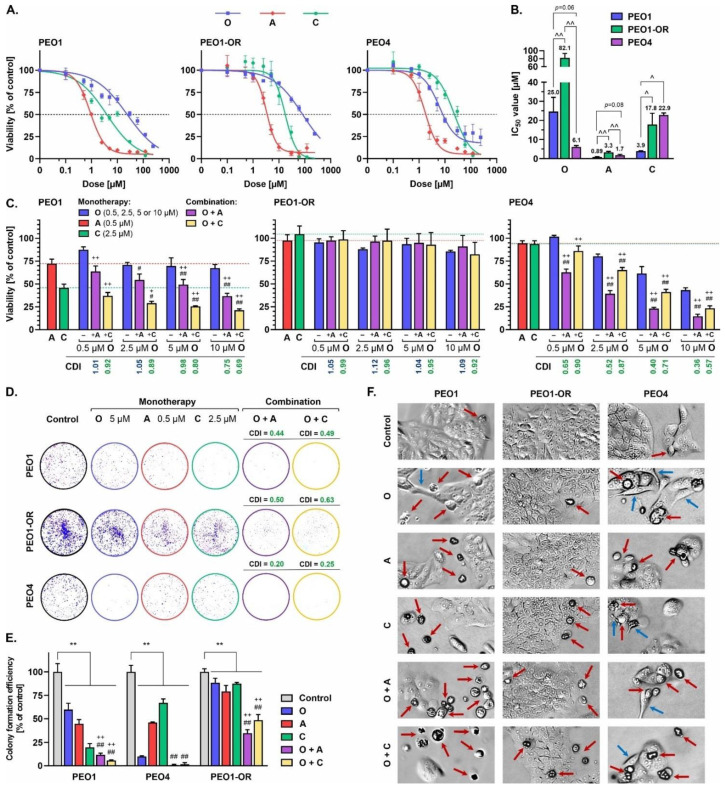
Olaparib combined with ATRi and CHK1i induces lower cytotoxicity in PEO1-OR cells compared to olaparib-sensitive cell lines. Cell viability in response to treatment for 5 days with olaparib (O, 0.1–240 μM), ATRi (A, 0.1–120 μM), and CHK1i (C, 0.1–120 μM) was determined using the MTT assay. (**A**) A four-parameter logistic regression model was fitted to dose–response data to estimate (**B**) absolute IC_50_ values. Data are expressed as mean ± SD (*n* ≥ 3). (**C**) Synergistic cytotoxic effects of drugs determined with the MTT assay in response to 5-day treatment with olaparib (0.5, 2.5, 5, or 10 μM) combined with ATRi (0.5 μM) or CHK1i (2.5 μM). Data are expressed as mean ± SD (*n* = 3–6). (**D**) The clonogenic assay was performed after incubation with olaparib (5 μM), ATRi (0.5 μM), CHK1i (2.5 μM), or their combinations for 5 days. After treatment, cells were allowed to grow and proliferate in the absence of drugs for 10–14 days. Representative images of colonies and (**E**) quantification of colony formation ability compared with control cells. Data are expressed as mean ± SD (*n* = 4). (**F**) Morphological changes of OC cell lines in response to olaparib (5 μM), ATRi (0.5 μM), CHK1i (2.5 μM), or their combinations for 5 days. Images were captured at 10× magnification. Coefficient of drug interaction (CDI) values indicating whether interaction effects are significantly synergistic (CDI < 0.7), synergistic (CDI < 1.0), additive (CDI = 1.0), or antagonistic (CDI > 1.0). Statistical significance was assessed using two-way ANOVA followed by Tukey’s test (IC_50_ values, viability, and colony formation efficiency). ^ *p* < 0.05, ^^ *p* < 0.01: significant differences between cell lines; ** *p* < 0.01: treatment vs. control; + *p* < 0.05, ++ *p* < 0.01: olaparib vs. combination with ATRi or CHK1i; # *p* < 0.05, ## *p* < 0.01: ATRi or CHK1i vs. respective combinations with olaparib.

Firstly, based on a dose–response curve of olaparib (0.1–240 μM) assessed over a 5-day treatment period in the parental PEO1 cell line, we determined the maximum suitable dose at the first stage of induction of resistance (10 μM) that led to significant inhibition of cell viability as assessed with the MTT assay (Figure 2A). Surviving cells were allowed to recover and re-populate during the passage, followed by retreatment with the selected dose, and further incrementally exposed to 2-fold higher doses of olaparib, up to a concentration of 80 μM. The entire population of remaining cells was subsequently cultured in the absence of the drug for a total of 72 days. Continuous treatment with increasing doses of olaparib resulted in a significant (3.3-fold) increase in drug resistance in PEO1-OR cells (IC_50_ = 82.1 μM) relative to sensitive PEO1 cells (IC_50_ = 25.0 μM) (Figure 2B), which may be considered clinically relevant [35]. Although we found a much higher IC_50_ value for olaparib in PEO1 cells with respect to those reported previously by our team [17], earlier findings collectively show various IC_50_ values ranging from at least 0.4 μM to 25 μM [19,36,37]. This apparent discrepancy can be attributed mainly to the reported subclonal evolution of PEO1 cells during passages resulting in the existence of two different subclones [38], the experimental design for the assay, the manufacturer of the drug, and the drug’s chemical purity.

In further biological studies, we used PEO1 and PEO4 cell lines as olaparib-sensitive cells and the PEO1-OR cell line as olaparib-resistant cells. PEO1 and PEO4 cell lines were originally derived at subsequent stages of treatment from ascites of serous OC patients [36] who carried the germline truncating *BRCA2* mutation. PEO4 was developed after disease progression following platinum-based chemotherapy [39] and displays a platinum-resistant phenotype [40]. Although sensitivity to platinum-based chemotherapy is predictive of the efficacy of response to olaparib in vitro and in vivo, suggesting similar mechanisms of resistance [41], the sensitivity of PEO4 cells to olaparib in comparison with OC cell lines with PARPi-induced resistance is ambiguous [19,36,42].

Further preliminary studies were conducted to estimate the proliferation rate and population doubling time (PDT) of PEO1, PEO1-OR, and PEO4 cell lines (Figure S1). All cells demonstrated a sigmoidal-like growth curve pattern typical for immortalized cell lines with a lag phase of about one day, followed by logarithmic growth and a stationary phase (Figure S1A). Notably, PEO1-OR cells began to recover and entered exponential growth as early as one day after plating. PDT estimates revealed the highest rate of proliferation in PEO1-OR cells (PDT = 31.9 h) and slower rates of division of PEO1 (PDT = 37.1 h) and PEO4 cells (PDT = 46.3 h) (Figure S1B) which were in line with original findings for olaparib-sensitive cells [39].

### 3.2. The PEO1-OR Cell Line Displays Increased Resistance to Olaparib and Inhibitors of the ATR/CHK1 Pathway

To evaluate the potential antitumor activities of olaparib, ATRi, CHK1i, and their combinations, the cytotoxic effects of the inhibitors in all OC cell lines were investigated using the MTT assay. Dose–response curves were generated to estimate the absolute IC_50_ values using a four-parameter logistic regression model following 5-day treatment of cells with increasing concentrations of olaparib (0.1–240 μM) and ATRi or CHK1i (0.1–120 μM) (Figure 2A). While a notable decrease in the survival of PEO1 cells was observed in response to olaparib at a broad range of drug concentrations (0.5–240 μM), the inhibitory influence of olaparib in PEO1-OR cells was markedly lower. Based on IC_50_ values for olaparib, PEO4 cells were more sensitive to PARPi than PEO1 and PEO1-OR cells (Figure 2B). However, at lower concentrations (0.1–2.5 μM), olaparib suppressed cell viability to a greater extent in PEO1 cells. PEO1-OR cells also displayed significantly reduced sensitivity to ATRi and CHK1i (IC_50_ = 3.3 μM and 17.8 μM, respectively) compared to the parental PEO1 cell line (IC_50_ = 0.89 μM and 3.9 μM, respectively) (Figure 2B).

We further examined the hypothesis that olaparib cytotoxicity could be potentiated in both sensitive and resistant OC cell lines by the concurrent addition of either ATRi or CHK1i promoting blockage of the ATR/CHK1 pathway [17,20,43]. Specific concentrations of olaparib (0.5, 2.5, 5, and 10 μM), ATRi (0.5 μM), and CHK1i (2.5 μM) were selected to determine the lowest doses conferring synergistic cytotoxicity across the olaparib-sensitive cell lines without significant reduction in viability of PEO1-OR cells (Figure 2C). The lowest dose of olaparib that exerted synergistic effects with ATRi or CHK1i in PEO1 and PEO4 was 5 μM. The addition of 0.5 μM ATRi markedly augmented the decrease in cell viability induced by 5 μM olaparib in PEO1 (CDI = 0.98) and PEO4 cells (CDI = 0.40), compared to PARPi alone. Synergistic effects were additionally observed upon co-administration of CHK1i with PARPi (0.5–10 μM) in olaparib-sensitive cell lines, where 2.5 μM CHK1i and 5 μM olaparib resulted in a marked decrease in cell viability (CDI = 0.80 for PEO1 and CDI = 0.71 for PEO4).

Simultaneously, monotherapy and other drug combinations had no significant effects on PEO1-OR metabolic activity, indicating that resistance to olaparib may also be correlated with decreased sensitivity to the inhibition of either ATR or CHK1 kinase. Based on data from the MTT assay, we selected 5 μM olaparib, 0.5 μM ATRi, and 2.5 μM CHK1i as the lowest doses that induced a synergistic decrease in viability of olaparib-sensitive cells for further experiments to compare OC cell responses to combined treatments regarding their distinct sensitivities to PARPi alone.

Next, we performed the clonogenic assay, a commonly used test to establish the long-term effects of cytotoxic agents on cell survival and proliferation [44]. Increased resistance of PEO1-OR cells to olaparib, ATRi, and CHK1i was observed relative to the olaparib-sensitive cell lines after 5 days of treatment (Figure 2D,E). Inhibition of PARP1 with concurrent blockage of ATR or CHK1 significantly decreased the ability of PEO1-OR cells to form colonies compared to untreated cells. Consistent with the results of the MTT assay (Figure 2C), olaparib-sensitive cells exhibited notably decreased survival in the presence of inhibitors, both alone and with their combinations (Figure 2D,E). The addition of ATRi or CHK1i to olaparib significantly reduced the colony formation efficiency in PEO1 cells (CDI = 0.44 and 0.49), PEO4 cells (CDI = 0.20 and 0.25), and PEO1-OR cells (CDI = 0.50 and 0.63).

Moreover, the morphology of PEO1 and PEO4 cells was altered in response to all the inhibitory agents tested after a 5-day incubation period as indicated by spherical cell shape, loose adherence, or detachment from the surface, frequently with cell fragmentation (indicated by red arrows) or cell elongation conferring a spindle-like shape (indicated by blue arrows) (Figure 2F). Additionally, monolayer confluency was affected after treatments in PEO1 and PEO4 cells. In contrast, no significant evidence of PEO1-OR cell deterioration was observed in the presence of olaparib or ATRi. The combination treatment affected the population of PEO1-OR cells less notably compared with PEO1 and PEO4 cells.

Taken together, the results indicate that PEO1-OR cells display decreased sensitivity to olaparib, along with cross-resistance to ATRi and CHK1i, relative to the parental cell line. Inhibition of the ATR/CHK1 pathway synergistically promotes the cytotoxicity of olaparib more notably in sensitive PEO1 and PEO4 cells. However, we confirmed the rationale for combined treatment in all OC cells.

### 3.3. Relationship between Olaparib Resistance and Exonic Variants in Genes Involved in DNA Damage Repair, Cell Cycle Regulation, and Drug Efflux in OC Cells

Whole-exome sequencing (WES) via NGS has been employed to identify functional mutations in OC to date [45,46]. Here, we investigated variants of genes encoding proteins involved in DNA damage repair, cell cycle regulation, and drug efflux (*ABCB1*, *ATR*, *BRCA1*, *BRCA2*, *CHEK1*, *H2AX*, *PARP1*, *PARG*, *RAD51*, *TP53*, *TP53BP1*) in PEO1 olaparib-sensitive and -resistant cell lines to identify putative genetic drivers of resistance to PARPi (Table 1 and Table S4). The average target coverage was 132× and 156× for PEO1 and PEO1-OR cells, respectively, providing a standard mean depth of coverage for clinical WES [47]. We achieved 96.3% and 96.7% breadth of coverage of the reference genome at a depth of 30× for PEO1 and PEO1-OR cells, respectively.

WES revealed 81 and 84 high-quality variants in PEO1 cells (exonic = 30, intronic = 51) and PEO1-OR cells (exonic = 30, intronic = 54), respectively, after filtering out low-quality variants as described in Section 2. Differences in intronic variants detected in PEO1 and PEO1-OR cells within intronic regions are presented in Table S4. We focused on the mutations detected in the exon regions crucial in maintaining the normal functions of encoded proteins. Sequencing data revealed that PEO1 and PEO1-OR cells harbored the same 30 exonic mutations in *ABCB1*, *ATR*, *BRCA1*, *BRCA2*, *CHEK1*, *H2AX*, *PARP1*, and *TP53* genes. Both cell lines displayed similar allele frequencies for most of these variants, indicating that the alterations had mostly no impact on the acquisition of resistance to olaparib. All variants involved a single nucleotide change, and 80% of these variants were located within the protein-coding sequences whereas alterations in untranslated regions (UTR) were less common. The highest numbers of alterations within protein-coding sequences were detected in *BRCA1* (four missense and three synonymous) and *BRCA2* (three synonymous, two missense, and one nonsense). Mutations resulting in a premature termination codon were found only in *BRCA2*, whereas synonymous (54%) and missense (42%) mutations accounted for most alterations detected. Among all 30 exonic variants, 27 were common in the general populations and 19 were classified as benign due to a lack of evidence for pathogenicity. Three alterations (two in *BRCA2* and one in *TP53*) located in a mutational hot spot and/or connected with previously reported data on pathogenicity (functional studies supportive of a damaging effect) were classified as pathogenic or of unknown significance (Table S1).

Regarding the impact of the variants on protein function, we identified three mutations predicted to cause loss of function (in *TP53*, *BRCA2*, and *PARP1*) and two causing gain of function (*ATR* and *CHEK1*). A pathogenic homozygous G244D (c.731G > A) mutation in *TP53* was present with 100% allele frequency in both OC cell lines, which is typical in 94–96% of HGSOC clinical cases [48,49]. Notably, *TP53*-mutated tumors are associated with abrogated G1/S cell cycle checkpoint and increased reliance on the G2/M checkpoint upon DNA damage [49]. While the identified *BRCA1* S1613G missense variant is localized in a mutational hot spot, functional assays have revealed no damaging effect on protein function [50,51]. Other *BRCA1* nonsynonymous variants (P871L, E1038G, K1183R) were classified as benign by the ClinVar variant database based on functional studies. Additionally, consistent with previous reports [52], BRCA1 proteins with each missense variant (P871L, E1038G, K1183R, S1613G) identified by our group possessed HR activity similar to that of the respective wild-type counterparts, as determined using the HR reporter assay. We additionally identified five SNVs in the *ATR* gene in both OC cell lines; however, they were classified as of benign pathogenicity due to high frequencies in general populations. Sequencing results showed that PEO1 and PEO1-OR possess two exonic SNVs of *CHEK1*, one located in a protein-coding sequence (c.1411A > G, p.I471V) and the other in the 3′ UTR (c.* 28–3033C > G). Both cell lines were homozygous for the missense variant *CHEK1* I471V, which was computationally predicted to cause gain-of-function activity. A comparison of the frequencies of alterations detected in PEO1 and PEO1-OR cell lines revealed significantly different allele frequencies of one mutation in *BRCA2* (34% vs. 94%) and one in *H2AX* (53% vs. 35%).

### 3.4. Upregulation of BRCA2 Is Correlated with a Secondary Reversion Mutation Accounting for Acquired Resistance to Olaparib in OC Cells

**Figure 3:**
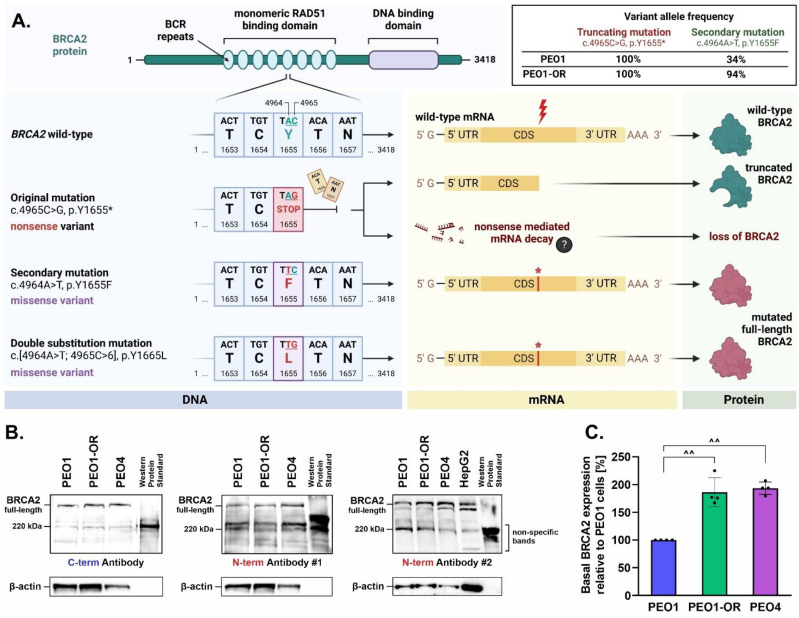
Restoration of wild-type BRCA2 open reading frame by secondary reversion mutation promotes upregulation of BRCA2, potentially accounting for olaparib resistance in PEO1-OR cells. (**A**) Schematic representation of the biochemical consequences of key BRCA2 variants detected using WES. PEO1-OR and PEO1 cells are homozygous for a truncating mutation (c.4965C > G, p.Y1655 *). Co-occurrence of a secondary mutation (c.[4964A > T; 4965C > G], p.Y1655L) removes the premature stop codon introduced by the truncation mutation originating from the PEO1 cell line, restoring the wild-type ORF of BRCA2. (**B**) Qualitative Western blot analysis of full-length and truncated BRCA2 in OC cells. The HepG2 cell line was used as a negative control of truncated BRCA2 expression to exclude non-specific bands. An anti-BRCA2 antibody against the C-terminus and two antibodies against the N-terminus (targeting different epitopes) were used to differentiate between the two protein forms. (**C**) Quantitative Western blot analysis of basal expression of full-length BRCA2 in OC using a monoclonal antibody against the N-terminus #2. The results are expressed as mean ± SD (*n* = 4). Statistical significance was assessed using ordinary one-way ANOVA followed by Tukey’s test. ^^ *p* < 0.01: differences between cell lines. CDS—coding sequence, UTR—untranslated region.

Intrinsic and acquired resistance to PARPi are associated with secondary reversion mutations in *BRCA1/2* genes that restore the open reading frame (ORF) in OC [40,53]. We confirmed that the PEO1 olaparib-sensitive cell line is a carrier of the *BRCA2* Y1655 * (c.4965C > G) variant (Table 1), consistent with previous reports as PEO1 cells were originally established from a female OC patient carrying the same mutation [40]. This pathogenic variant was predicted to result in a termination signal at position 1655 resulting in a truncated protein product in case of successful translation of mRNA (Figure 3A). The same SNV was detected in the PEO1-OR cell line, with 100% allele frequency in both cases. Within the same nucleotide triplet (TAC) at position 1655 in wild-type *BRCA2*, we identified a secondary single-nucleotide substitution (c.4964A > T, p.Y1655F) in both PEO1 and PEO1-OR cells. Notably, PEO1-OR cells were enriched (allele frequency of 94%) in the c.4964A > T variant compared to parental PEO1 cells (allele frequency of 34%). Interestingly, the c.4964A > T variant could be considered a reversion mutation, as the co-occurrence of both adjacent SNVs (c.4964A > T and c.4965C > G) may reestablish the ORF of *BRCA2* by altering a stop codon (TAG) to a nucleotide triplet encoding Leu (TTG).

Next, we biochemically confirmed via Western blot that PEO1 and PEO1-OR cells restored the ORF of *BRCA2* expressing full-length protein (Figure 3B). PEO4 cells with a known *BRCA2* reversion mutation (c.4965C > T, p.Y1655Y) and HepG2 cells were used as negative controls of truncated BRCA2 expression [40]. BRCA2 protein was detected in PEO1, PEO1-OR, and PEO4 cells using antibodies recognizing C-terminal and N-terminal regions, indicating that all the OC cell lines express full-length BRCA2 (Figure 3B). Surprisingly, the truncated BRCA2 variant (Y1655 *) was not detected in PEO1 cells using two different anti-BRCA2 (N-term) antibodies (Figure 3B), suggesting that expression of the variant possessing a single truncation mutation is abrogated in PEO1 cells, which was further confirmed using SKOV-3 and OV-90 cells as negative controls (Figure S2). Interestingly, quantitative analysis of basal intact BRCA2 expression revealed significant upregulation of BRCA2 levels in PEO1-OR cells compared to PEO1 cells (Figure 3C). Hence, we hypothesize that subclonal selection of the *BRCA2* reversion variant contributes to the overexpression of the protein in olaparib-resistant PEO1-OR cells.

### 3.5. Inhibition of MDR1 Drug Efflux Pump Does Not Restore Sensitivity to Olaparib, ATRi, or CHK1i in Olaparib-Resistant Cells

The MDR1 efflux pump is associated with resistance to olaparib in OC cells [54]. Hence, we examined the potential involvement of MDR1 in the cytotoxic activity of olaparib, ATRi, CHK1i, and combination treatments in HGSOC cell lines.

**Figure 4:**
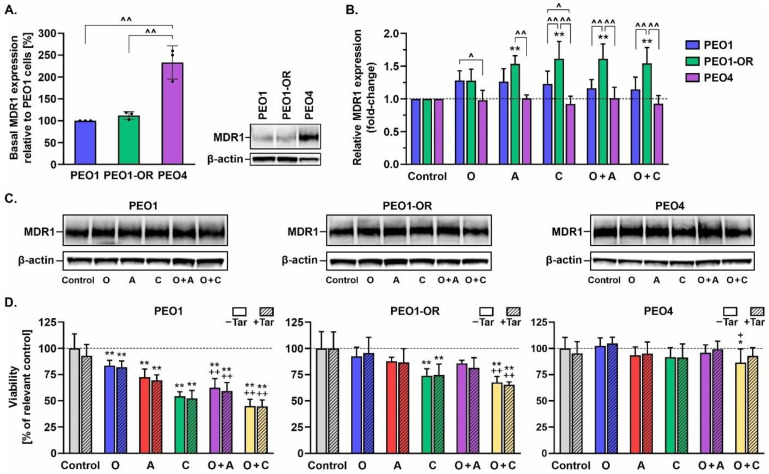
MDR1 efflux pump makes a negligible contribution to the sensitivity of HGSOC cell lines to olaparib, ATRi, and CHK1i. (**A**) Quantitative Western blot analysis of basal MDR1 expression in untreated OC cell lines cultured for 72 h and (**B**) changes in MDR1 after incubation with the inhibitor compounds for 48 h. Protein levels were quantified following normalization to β-actin and calculated as fold-change relative to untreated controls. Basal levels of protein were calculated relative to PEO1 cells. Results are expressed as mean ± SD (*n* = 3). Statistical significance was assessed using ordinary one-way ANOVA (basal protein expression) and two-way ANOVA (responses to tested compounds), both followed by Tukey’s test. ^ *p* < 0.05, ^^ *p* < 0.01: differences between cell lines; * *p* < 0.05, ** *p* < 0.01: treatment vs. control; + *p* < 0.05, ++ *p* < 0.01: olaparib vs. combinations with ATRi or CHK1i. (**C**) Representative Western blot images. (**D**) Cell viability, defined as cellular metabolic activity, was assessed using the MTT assay in response to treatment for 2 days with olaparib (O, 5 μM), ATRi (A, 0.5 μM), CHK1i (C, 2.5 μM), or their combinations for 48 h in the absence and presence of the MDR1 inhibitor tariquidar (Tar, 0.1 μM). Data are expressed as mean ± SD (*n* = 3). Ordinary one-way ANOVA followed by Tukey’s test or Brown–Forsythe version of one-way ANOVA followed by Dunnett’s T3 test was used to compare the sensitivity of the cell lines to the test compounds.

We observed no significant differences in the basal expression of MDR1 between PEO1 and PEO1-OR cell lines (Figure 4A). Interestingly, a 2.3-fold increase in MDR1 expression was evident in untreated PEO4 compared to PEO1 cells. Olaparib had no significant effect on MDR1 expression in OC cells after 48 h of treatment (Figure 4B). However, PEO1-OR cells with decreased sensitivity to tested inhibitors showed a significant upregulation of MDR1 after incubation with ATRi, CHK1i, or their combinations with PARPi. Next, we assessed the viability of OC cells in response to the drugs after 48 h in the presence of a non-toxic concentration (0.1 μM) of a specific inhibitor of MDR1, tariquidar [55], using the MTT assay (Figure 4D). Simultaneous incubation with tariquidar and the above inhibitor compounds did not significantly affect the viability of OC cell lines after 48 h relative to treatment with the drugs in the absence of the MDR1 inhibitor. Blockage of MDR1 in PEO1-OR cells did not significantly restore the augmented sensitivity of parental PEO1 cells to olaparib, ATRi, and CHK1i (Figure 4D). To further clarify the role of MDR1, we employed doxorubicin (an MDR1 substrate) and P-glycoprotein inhibitors (tariquidar and verapamil) in an MDR1-expressing HepG2 cell model of drug transport (Figure S3). The addition of verapamil (50 μM) or tariquidar (0.1 μM) to doxorubicin led to significantly enhanced cytotoxicity in HepG2 cells in a dose-dependent manner, compared to doxorubicin alone, indicating that verapamil and tariquidar inhibit MDR1 activity. Notably, however, inhibition of MDR1 with either verapamil or tariquidar alone did not enhance the response of HepG2 cells to olaparib, ATRi, CHK1i, or their combinations (Figure S3).

### 3.6. Olaparib Synergistically Increases Cleavage of PARP1 in Olaparib-Sensitive OC Cells in Combination with ATRi or CHK1i

**Figure 5:**
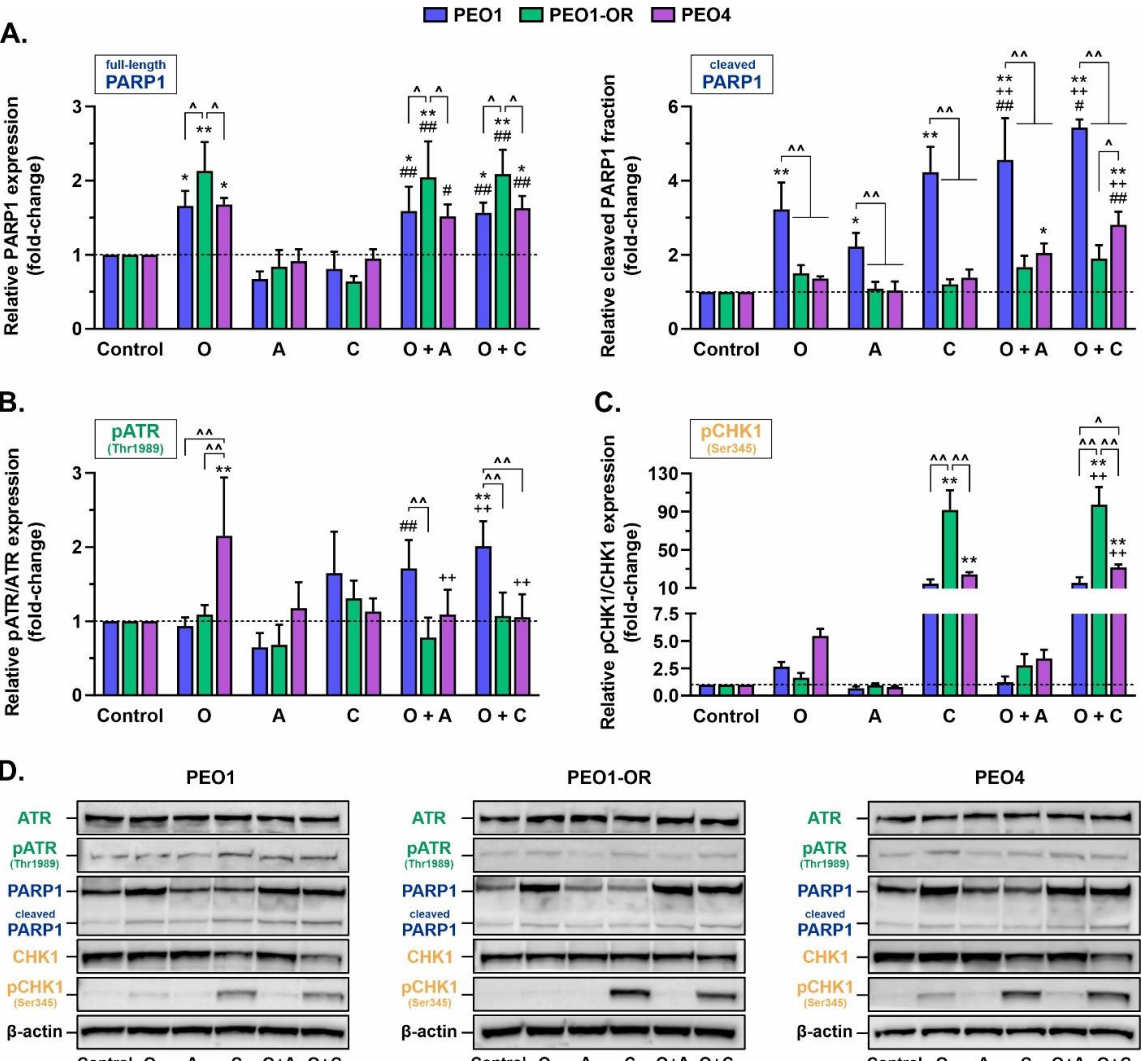
Monotherapy with olaparib, ATRi, and CHK1i or their combinations alter the expression and phosphorylation levels of targeted proteins and PARP1 cleavage in a cell-dependent manner. Quantitative Western blot analysis of (**A**) full-length and cleaved PARP1, (**B**) total and phosphorylated ATR (Thr1989), and (**C**) total and phosphorylated CHK1 (Ser345) in OC cell lines. Cells were incubated with olaparib (O, 5 μM), ATRi (A, 0.5 μM), CHK1i (C, 2.5 μM), or their combinations for 48 h, and whole-cell lysates were prepared immediately afterward. Protein levels were quantified following normalization to β-actin and calculated as fold-change relative to untreated controls. The results are presented as mean ± SD (*n* = 3). Statistical significance was assessed using two-way ANOVA followed by Tukey’s test. ^ *p* < 0.05, ^^ *p* < 0.01: differences between cell lines; * *p* < 0.05, ** *p* < 0.01: treatment vs. control; ++ *p* < 0.01: olaparib vs. combinations with ATRi or CHK1i; # *p* < 0.05, ## *p* < 0.01: ATRi or CHK1i vs. respective combinations with olaparib. (**D**) Representative Western blot images.

Next, we evaluated the mechanisms underlying the synergistic cytotoxicity of olaparib combined with ATRi or CHK1i. Basal levels of PARP1 were similar in all OC cell lines (Figure S4). The expression of PARP1 was significantly increased in all OC cells treated for 48 h with olaparib alone compared to control cells (Figure 5A); however, PEO1-OR cells exhibited the highest elevation of PARP1 expression in response to olaparib (by 2.1-fold). Compared to control cells, the addition of either ATRi or CHK1i to olaparib led to significant upregulation of PARP1, but to the same level as that observed with PARPi monotherapy. Olaparib, ATRi, and CHK1i alone induced marked elevation of the cleaved PARP1 fraction, a biomarker of caspase-dependent apoptosis [53,54], compared with the control group in PEO1 cells only (by 3.2-fold and 4.2-fold, respectively). PARPi also induced a significant synergistic increase in cleaved PARP1 compared to the control group in the presence of ATRi or CHK1 only in olaparib-sensitive cells, in line with the results of the MTT assay (Figure 2C).

### 3.7. PEO1-OR Cells Bypass Olaparib-Induced Activation of the ATR/CHK1 Pathway

We speculated that increased G2/M checkpoint arrest in OC cell lines is primarily activated through ATR/CHK1 signaling based on the finding that olaparib-sensitive cells display increased reliance on the ATR/CHK1 axis [17,23]. To examine this hypothesis, we investigated the phosphorylation of ATR at Thr1989 and CHK1 at Ser345, events responsible for the activation of ATR/CHK1 signaling in response to DNA damage [26,27].

As shown in Figure 5, olaparib treatment had a significant impact on the ATR/CHK1 pathway. Auto-phosphorylation of ATR was significantly induced in PEO4 cells by 2.1-fold (Figure 5B), and notably increased phosphorylation of pCHK1 was observed in PEO1 and PEO4 cells by 2.7- and 5.5-fold, respectively (Figure 5C), suggesting activation of ATR/CHK1 signaling for survival. The addition of ATRi decreased PARPi-induced phosphorylation of pCHK1 in PEO1 and PEO4 cells, indicating inhibition of ATR activation. As expected [56,57], the inhibition of CHK1 caused the accumulation of CHK1 phosphorylated at Ser345 in all OC cell lines. On the contrary, olaparib alone or combined with ATRi only non-significantly increased levels of pCHK1 (by 2.8-fold) irrespective of ATR phosphorylation in PEO1-OR cells, indicating decreased reliance on ATR/CHK1 signaling for survival in olaparib-resistant cells.

### 3.8. Olaparib Combined with Blockage of ATR/CHK1 Enhances DNA Double-Strand Breaks Irrespective of RAD51-Mediated HR Activity in BRCA2^MUT^ Ovarian Cancer Cells

PARPi agents promote the accumulation of DSBs in OC cells [17,23], resulting in phosphorylation of H2AX at serine 139, whereas RAD51 recombinase is a critical factor involved in the repair of deleterious DSBs by HR, serving as a useful marker for HR proficiency and recruitment of HR repair factors [9,58]. We hypothesized that inhibition of PARP1 and blockage of the ATR/CHK1 pathway could trigger elevated phosphorylation of H2AX, with the significant formation of γH2AX only in olaparib-sensitive cells.

**Figure 6:**
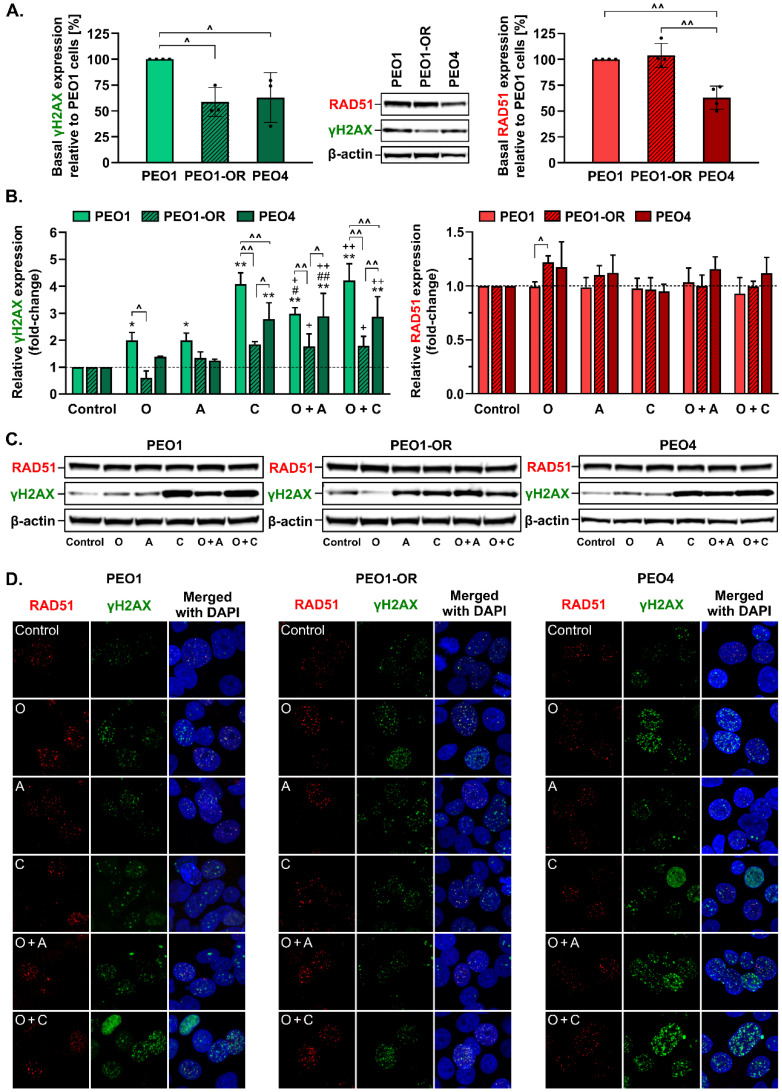
Olaparib-resistant cells exhibit decreased phosphorylation of the DSB marker H2AX in response to inhibitor treatment compared to sensitive OC cell lines. Quantitative Western blot of (**A**) RAD51 and (**B**) phosphorylation of H2AX at Ser139 to γH2AX in OC cell lines. Basal protein expression calculated from densitometric data relative to PEO1 cells. Cells were incubated for 48 h with olaparib (O, 5 μM), ATRi (A, 0.5 μM), CHK1i (C, 2.5 μM), or their combinations, and whole-cell lysates prepared immediately afterward. Protein levels following treatment were quantified, with normalization to β-actin as a loading control, and calculated as fold-change relative to untreated controls. The results are presented as mean ± SD (*n* = 3). Statistical significance was assessed using ordinary one-way ANOVA (basal protein expression) and two-way ANOVA (responses to tested compounds), both followed by Tukey’s test. ^ *p* < 0.05, ^^ *p* < 0.01: differences between cell lines; * *p* < 0.05, ** *p* < 0.01: treatment vs. control; + *p* < 0.05, ++ *p* < 0.01: olaparib vs. combinations with ATRi or CHK1i; # *p* < 0.05, ## *p* < 0.01: ATRi or CHK1i vs. respective combinations with olaparib. (**C**) Representative Western blot images of RAD51 and γH2AX. (**D**) Representative immunofluorescence images at 63× oil magnification of co-localized RAD51 foci (red) with γH2AX foci (green) and nuclei stained with DAPI (blue) in OC cells exposed to the combined inhibitor compounds.

The basal phosphorylation level of γH2AX was significantly higher in untreated PEO1 cells compared to PEO1-OR and PEO4 cells (Figure 6A). Only PEO1 cells exhibited significant changes in γH2AX expression in response to all the inhibitor compounds after 48 h (Figure 6B). Upon treatment with olaparib or ATRi, similar increases in phosphorylation of H2AX were observed (by 2.0-fold), whereas CHK1i induced a 4.1-fold change in γH2AX. Combined treatment with olaparib and ATRi synergistically augmented γH2AX levels in olaparib-sensitive cells, indicating that olaparib-sensitive cells were the most susceptible to DNA damage. Interestingly, γH2AX was increased to similar levels in the presence of CHK1i alone or combined with olaparib in all OC cells. Similar to data obtained with olaparib and ATRi, PEO1-OR cells exhibited the lowest elevation of γH2AX upon co-incubation with olaparib and CHK1i (1.8-fold) relative to PEO1 (4.2-fold) and PEO4 (2.9-fold) cells. Basal RAD51 expression was comparable in untreated PEO1 and PEO1-OR cells but significantly downregulated in PEO4 cells (Figure 6B). No significant changes in RAD51 protein levels were detected in all OC cell lines exposed to the inhibitor compounds compared to untreated cells. Following olaparib treatment, PEO1-OR cells exhibited moderately increased expression of RAD51 compared to parental PEO1 cells (Figure 6B), which may be associated with resistance to PARPi.

Recruitment of RAD51 to DNA lesion sites has been proposed as a biomarker of HR proficiency in ovarian cancer cells [59]. Furthermore, we confirmed that the BRCA2 variant (c.[4964A > T; 4965C > G], p.Y1655L) harboring a missense mutation in a BRC5 repeat responsible for RAD51 binding was functional and RAD51 recruitment to DNA damage sites was not disrupted in *BRCA2*^MUT^ OC cells by monitoring formation and co-localization of RAD51 and γH2AX foci in OC cells (Figure 6D). RAD51 formed foci at the sites of DSBs, represented by γH2AX foci, in all OC cell lines, suggesting that *BRCA2*^MUT^ OC cells are HR-proficient. We observed the most apparent γH2AX foci formation in PEO1 and PEO4 cells in response to olaparib combined with CHK1i (Figure 6D). In PEO4 and PEO1-OR cells, a major fraction of γH2AX foci co-localized with RAD51 foci with each treatment. Lower levels of phosphorylated H2AX and reduced γH2AX foci formation in PEO1-OR cells compared to PEO1 and PEO4 cells in response to studied inhibitors confirm the phenotype of decreased drug sensitivity in olaparib-resistant cells.

### 3.9. Olaparib-Resistant Cells Exhibit Decreased Accumulation of Chromosomal Aberrations and DNA Double-Strand Breaks Compared to Sensitive OC Cell Lines

**Figure 7:**
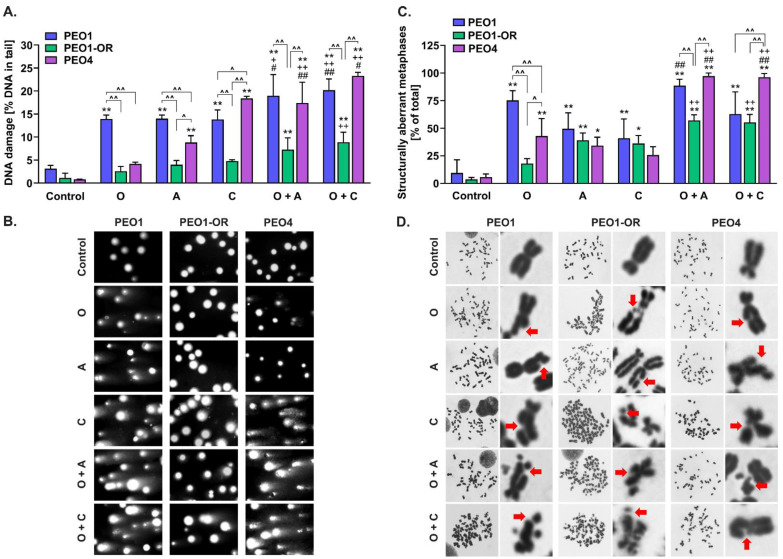
Resistance to olaparib is correlated with reduced accumulation of DSBs, generating fewer structural chromosomal aberrations in PEO1-OR cells. (**A**) DNA double-strand breaks examined with the neutral comet assay in OC cells exposed to olaparib (5 μM), ATRi (0.5 μM), CHK1i (2.5 μM), or their combinations for 48 h. The DNA strand-breaking agent doxorubicin (2.5 μM) was used as a positive control in the comet assay. Results are expressed as mean ± SD (*n* = 4). (**B**) Representative images of DNA comets with tails. (**C**) Structural metaphase chromosomal aberrations induced by inhibitors in OC cells. Cells were incubated with olaparib (O, 5 μM), ATRi (A, 0.5 μM), CHK1i (C, 2.5 μM), or their combinations for 48 h and arrested at the mitosis stage in the presence of colcemid (0.1 μg/mL) for 1 h. Metaphase chromosomes were stained with Giemsa and visualized at 100× magnification. Chromosomal structural aberrations were analyzed in 105 metaphase spreads scored for abnormalities for each group. Results are expressed as mean ± SD (*n* = 3). Statistical significance was assessed using two-way ANOVA followed by Tukey’s test. ^ *p* < 0.05, ^^ *p* < 0.01: differences between cell lines; * *p* < 0.05, ** *p* < 0.01: treatment vs. control; + *p* < 0.05, ++ *p* < 0.01: olaparib vs. combinations with ATRi or CHK1i; # *p* < 0.05, ## *p* < 0.01: ATRi or CHK1i vs. respective combinations with olaparib. (**D**) Representative images of metaphase spreads with aberrant chromosomes indicated by red arrows (chromatid gaps and breaks).

DNA DSBs are pivotal lesions leading to the formation of structural chromosomal aberrations and HRD-associated genomic instability in OC cells [60,61,62]. The DNA lesion levels evaluated in studied HGSOC cell lines after 48 h of incubation (Figure 7) were correlated with the cytotoxic activity of inhibitors after 5 days, as determined with the MTT assay (Figure 2), and mostly with γH2AX expression (Figure 6B).

The results of the neutral comet assay showed significantly increased comet tail formation after treatment with all the inhibitors in PEO1 compared to PEO1-OR cells (Figure 7A,B), indicating decreased DNA damage in olaparib-resistant cells. Treatment of PEO1-OR and PEO4 with PARPi did not induce elevation of DSBs, which were significantly higher in PEO1 cells by 5.4-fold and 3.3-fold, respectively. Olaparib, ATRi, and CHK1i exerted no significant effects on DSB levels in PEO1-OR cells, while a synergistic genotoxic effect of combined treatments was observed in PEO1 and PEO4 cells. In PEO1 cells, the addition of ATRi or CHK1i to olaparib significantly augmented PARPi-induced comet tail formation from 14.0% to 19.0% and 20.2%, respectively. Moreover, combined treatment sensitized PEO4 cells to olaparib, causing a marked increase in DSB formation to levels comparable to those of PEO1 cells (Figure 7A).

We further analyzed structural chromosomal abnormalities induced by the inhibitors after 48 h in OC cell lines (Figure 7C,D). Olaparib exerted more significant structural abnormalities in PEO1 cells than in PEO1-OR and PEO4 cells (Figure 7C), in line with marked olaparib-induced accumulation of DSBs (Figure 7A). Blockage of the ATR/CHK1 pathway did not cause a significant increase in tail DNA in PEO1-OR cells (Figure 7A), suggesting that ATRi- and CHK1-induced chromosomal instability in olaparib-resistant cells is attributable to other types of DNA lesions (Figure 7C). Combination of olaparib with ATRi or CHK1i synergistically elevated olaparib-induced chromosomal alterations in PEO4 cells only, as evident from the marked increase in the aberrant metaphase (up to 97% and 96%, respectively). Notably, the addition of ATRi or CHK1i to olaparib caused a less prominent elevation in aberrant metaphase in PEO1-OR (by 32% and 8% for O + A and O + C, respectively) and PEO4 cells (by 40% for O + A and 41% O + C, respectively) relative to PEO1 cells (Figure 7C).

Our results collectively showed significantly lower levels of DSBs and chromosomal aberrations in olaparib-resistant cells compared to PEO1 and PEO4 cells. Enhanced generation of DSBs in response to the combination of olaparib with ATRi or CHK1i in olaparib-sensitive cells was correlated with increased accumulation of aberrant metaphases, indicating genotoxic instability in OC cells.

### 3.10. PEO1-OR Cells Progress Normally through the Cell Cycle upon Inhibition of PARP1 and the ATR/CHK1 Axis

**Figure 8:**
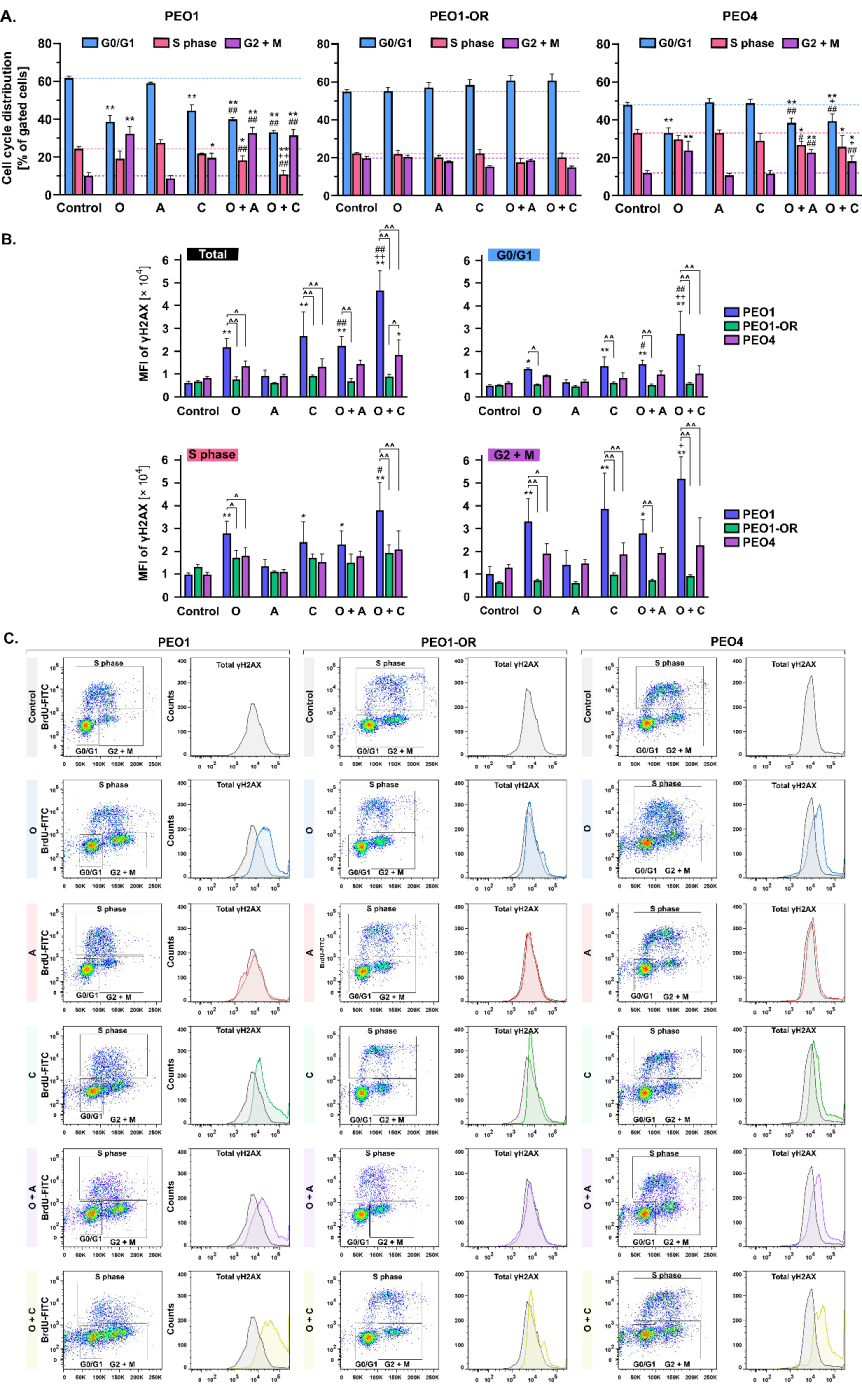
Effects of olaparib, ATRi, CHK1i, and their combinations on cell cycle distribution and accumulation of γH2AX depend on the sensitivity of OC cell lines to olaparib. (**A**) Cell cycle analysis with concurrent (**B**) quantitative assessment of γH2AX levels was performed via flow cytometry. Briefly, cells were incubated for 48 h with olaparib (O, 5 μM), ATRi (A, 0.5 μM), CHK1i (C, 2.5 μM), or their combinations, pulsed with BrdU during the final 3 h of the treatment, and harvested for co-staining of incorporated BrdU and intracellular γH2AX. Levels of γH2AX were assessed based on geometric mean fluorescence intensity (MFI). Results are expressed as mean ± SD (*n* = 3). Statistical significance was assessed using two-way ANOVA followed by Tukey’s test. ^ *p* < 0.05, ^^ *p* < 0.01: differences between cell lines; * *p* < 0.05, ** *p* < 0.01: treatment vs. control; + *p* < 0.05, ++ *p* < 0.01: olaparib vs. combinations with ATRi or CHK1i; # *p* < 0.05, ## *p* < 0.01: ATRi or CHK1i vs. respective combinations with olaparib. (**C**) Dot plots of BrdU/FITC and 7-AAD stained cells and histograms with overlay signals of PE-conjugated anti-γH2AX antibody from untreated (gray) and treated (color) cells.

Treatment with olaparib is associated with extended arrest at either the G0/G1 [63] or G2/M phase [64] of the cell cycle in OC cell lines. Therefore, we concurrently evaluated the effects of inhibitors tested on cell cycle distribution and DSB accumulation (Figure 8).

As expected, following 48 h incubation, olaparib induced a G2/M block in PEO1 cells compared to untreated cells where the G2/M phase subpopulation increased from 10% to 32%, accompanied by a 23% decrease in the G0/G1 phase subpopulation (Figure 8A). However, the addition of ATRi or CHK1i had an effect similar to that of PARPi alone on G2/M arrest in PEO1 cells. Analogous to PEO1 cells, olaparib alone and combined with ATRi or CHK1i induced notable G2/M arrest in PEO4 cells. Interestingly, olaparib-resistant cells progressed normally through all phases of the cell cycle following treatment with the inhibitors, both alone and in combination (Figure 8A).

Simultaneously, we did not observe significant changes in phosphorylation of H2AX in PEO1-OR cells treated with either single or combined inhibitors in contrast to parent PEO1 cells (Figure 8), which correlated with analysis of γH2AX by Western blot (Figure 6B). Phosphorylation of H2AX increased substantially in all subpopulations of PEO1 cells in response to olaparib (by 3.6-fold), CHK1i (by 4.5-fold), and the combination of olaparib with either ATRi (by 3.7-fold) or CHK1i (by 7.8-fold) relative to untreated cells, indicating that H2AX phosphorylation occurs throughout the entire cell cycle. Interestingly, co-treatment of PEO1 cells with olaparib and CHK1i synergistically promoted the γH2AX level compared to monotherapies. In all subpopulations of PEO4 cells, significant changes in γH2AX levels were detected only upon co-treatment with olaparib and CHK1i compared to untreated cells, where S and G2/M phases predominantly contributed to the increase in γH2AX levels (Figure 8).

Overall, the distribution of PEO1-OR cells in the cell cycle was relatively unchanged in response to the inhibitor treatments. Moreover, combined treatment with olaparib and blockers of the ATR/CHK1 axis led to G2/M phase arrest in olaparib-sensitive cells.

### 3.11. Crosstalk between BRCA1 and 53BP1 as an Indicator of Sensitivity to Olaparib and ATR/CHK1 Pathway Blockers

**Figure 9:**
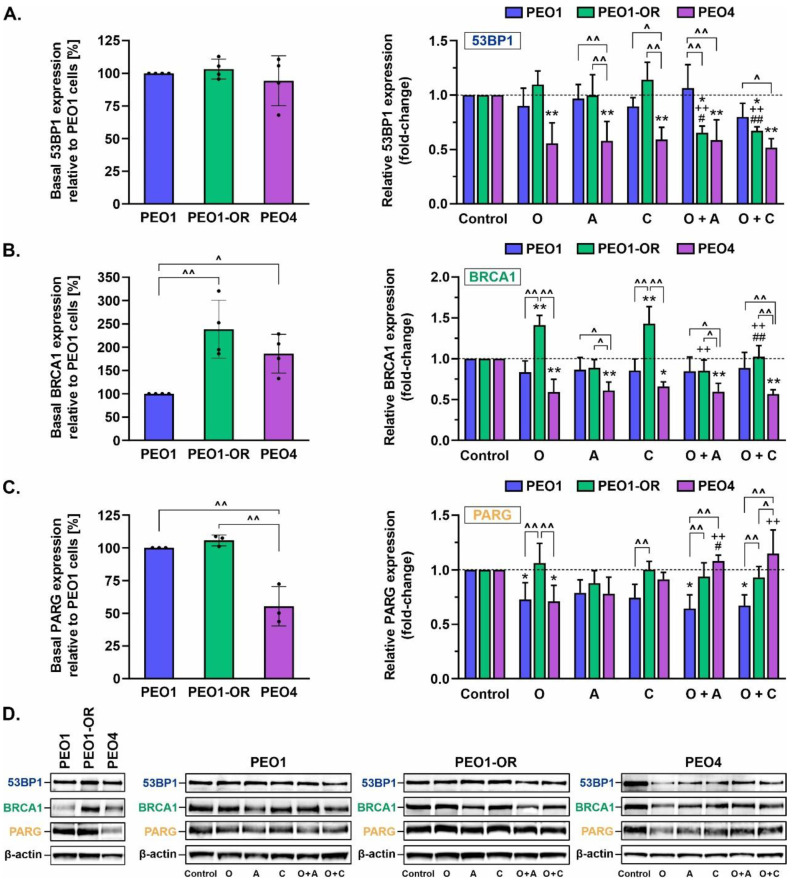
Changes in 53BP1, BRCA1, and PARG expression in OC cell lines treated with olaparib or inhibitors of the ATR/CHK1 pathway. Quantitative Western blot analysis was performed to assess basal expression of (**A**) 53BP1, (**B**) BRCA1, and (**C**) PARG. Protein levels upon treatment were quantified following normalization to β-actin as a loading control and calculated as fold-change relative to untreated controls. Cells were treated for 48 h with olaparib (O, 5 μM), ATRi (A, 0.5 μM), CHK1i (C, 2.5 μM), or their combinations, and whole-cell lysates were prepared immediately afterward. Data are expressed as mean ± SD (*n* = 3–4). Statistical significance was assessed using ordinary one-way ANOVA (basal protein expression) and two-way ANOVA (responses to tested compounds), both followed by Tukey’s test. ^ *p* < 0.05, ^^ *p* < 0.01: differences between cell lines; * *p* < 0.05, ** *p* < 0.01: treatment vs. control; ++ *p* < 0.01: olaparib vs. combinations with ATRi or CHK1i; # *p* < 0.05, ## *p* < 0.01: ATRi or CHK1i vs. respective combinations with olaparib. (**D**) Representative Western blot images.

HR repair may be reactivated and potentially drive resistance to PARPi by reacquisition of DNA end resection via loss of 53BP1 in *BRCA1*-depleted cells [5]. Therefore, we examined the expression levels of BRCA1 (mediating RAD51 loading and HR-dependent DSB repair) and 53BP1 (promoting NHEJ-dependent repair) via Western blotting after 48 h of incubation with studied inhibitors (Figure 9).

Our data showed that basal levels of 53BP1 protein were similar across all HGSOC cell lines (Figure 9A). Moreover, basal BRCA1 expression was significantly upregulated in untreated PEO1-OR and PEO4 cells by 2.4-fold and 1.9-fold, respectively, compared to PEO1 cells (Figure 9B). In PEO1-OR cells, single treatment with olaparib and CHK1i induced a significant increase in BRCA1 expression by about 1.4-fold compared to untreated cells, with no concomitant changes in 53BP1 levels. Interestingly, a combination of both drugs induced the opposite response in PEO1-OR cells. Indeed, the addition of CHK1i to olaparib reduced BRCA1 expression to control levels and synergistically reduced expression of 53BP1 by 38% to a level similar to that in PEO4 cells. However, ATRi had no influence on 53BP1 and BRCA1 expression in PEO1-OR cells, and combined treatment with olaparib and ATRi exerted an effect similar to that of olaparib and CHK1i. In PEO4 cells, incubation with single inhibitors and their combinations caused a significant decrease in BRCA1 expression to similar levels (by ~34–43%), along with 53BP1 (by ~41–48%), compared to untreated cells.

### 3.12. Olaparib Alone and Combined with Blockage of the ATR/CHK1 Pathway Suppresses PARG Expression in PEO1 Olaparib-Sensitive OC Cells

Next, we examined protein levels of poly(ADP-ribose) glycohydrolase (PARG) in response to inhibitor treatment (Figure 9C), as its loss is associated with PARPi resistance through suppression of olaparib-induced abrogation of PARP signaling and DNA damage [65].

Basal levels of PARG were unchanged among PEO1-OR and parental PEO1 cells. Notably, a significant reduction in basal PARG expression (by ~45%) was observed in PEO4 cells relative to the other two cell lines. The inhibitors had no significant effects on PARG levels in PEO1-OR cells; however, olaparib induced a decrease in PARG levels by 27% and 29% compared to untreated PEO1 and PEO4 cell lines, respectively. In PEO1 cells, the combination of olaparib with ATRi or CHK1i resulted in a similar decline in PARG levels to that observed upon PARPi treatment alone. PARG was restored to the basal level observed in untreated PEO4 cells upon the addition of ATRi or CHK1i. Interestingly, partial loss of PARG in PEO1 olaparib-sensitive cells accompanied upregulation of PARP1 in response to olaparib alone and in combination with ATRi and CHK1i (Figure 5A). Combined treatment with olaparib and ATRi and CHK1i also upregulated PARP1 in PEO1-OR and PEO4 cells, as shown previously (Figure 5A), but induced no significant changes in PARG levels (Figure 9C).

## 4. Discussion

Increasingly frequent clinical usage of PARPi inevitably leads to the emergence of resistance, constituting a growing problem for OC patients who relapse after treatment. Interestingly, prior exposure to PARPi is reported to decrease response to subsequent platinum-based chemotherapy in OC patients [2], which limits further therapeutic options. Over the past decade, numerous intracellular events that confer resistance to PARPi have been uncovered, including HR-dependent and HR-independent mechanisms, which are not mutually exclusive [11,16]. However, findings to date are insufficient to establish the mechanisms underlying the resensitization of OC cells to PARPi. Here we investigated how *BRCA2*^MUT^ HGSOC cells confer resistance to olaparib and the therapeutic benefits of combined treatment with PARPi and inhibitors of the ATR/CHK1 pathway in olaparib-sensitive and -resistant cells in vitro. Furthermore, we compared cellular and molecular responses to studied treatments, with the aim of developing a promising strategy to sensitize OC to olaparib [16,17,22,23,66].

Firstly, we successfully developed a PEO1-OR olaparib-resistant cell line from PEO1 HGSOC cells harboring deleterious *BRCA2* mutations as novel OC models displaying the PARPi-resistant phenotype are indispensable for the elucidation of cellular events that contribute to a compromised response to olaparib [40]. The PEO1-OR cell model was established in vitro using moderate, stepwise increasing doses of olaparib to select stable cells with decreased sensitivity to PARPi. Considering the selection strategy and 3.3-fold decrease in olaparib cytotoxicity (according to IC_50_ values), the newly developed cell line was classified as a clinically relevant model [35]. Data from the clonogenic assay confirmed significantly increased resistance of PEO1-OR cells to olaparib.

**Figure 10:**
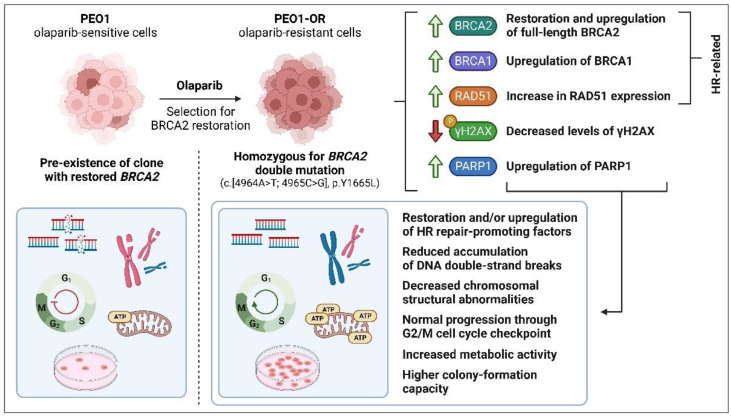
Co-existing mechanisms conferring resistance to olaparib in PEO1-OR *BRCA2*^MUT^ HGSOC cells. PEO1-OR cells underwent selection for BRCA2 restoration following prolonged treatment with olaparib, which led to enrichment in sub-dominant clones present in parental cells harboring the *BRCA2* double mutation (c.[4964A > T; 4965C > G], p.Y1655L) removing the premature termination codon. Upregulation of BRCA2, BRCA1, RAD51, and PARP1, along with a concurrent decrease in γH2AX levels, contributed to decreased sensitivity to olaparib.

Following the confirmation of olaparib resistance, we showed that PEO1-OR cells acquire resistance following prolonged treatment with olaparib through multiple co-existing mechanisms (Figure 10). Data from WES analysis showed that PEO1-OR cells are enriched for a double mutation (c.[4964A > T; 4965C > G], p.Y1655L) restoring functional full-length BRCA2 protein, suggesting that PARPi promoted subclonal selection for the variant present in a small subpopulation of parental PEO1 cells. This observation is in line with the finding of *BRCA2* reversion mutations after treatment of OC patients with PARPi [41,53,67]. However, previous works have demonstrated that PEO1 cells may acquire resistance to olaparib with or without restoration of BRCA2 [68,69], which indicates that conferring resistant phenotype in *BRCA2*^MUT^ HGSOC cells can occur in various ways. Moreover, Sakai et al. [40] showed that the selection of PEO1 cells with cisplatin may also induce the secondary mutation (4964A > T) restoring the *BRCA2* ORF, which is crucial for acquired resistance to chemotherapy. Unexpectedly, we did not detect expression of the truncated BRCA2 protein in PEO1 cells, which could be attributed to nonsense-mediated mRNA decay of *BRCA2* gene transcripts with a premature stop codon, a cellular mechanism that prevents expression of truncated proteins [70]. Our findings are consistent with previous studies reporting the occurrence of a secondary *BRCA2* mutation and the presence of full-length BRCA2 in PEO1 cells [71,72]. Interestingly, various variants of BRCA2 have been detected in different stocks of PEO1 cells, which has been already extensively discussed [38,72]. We speculate that divergent genetic alterations of BRCA2 may affect the expression of the full-length protein. As reported by Ciucci et al. [73], cells with *BRCA2* deficiency display decreased survival in vitro, which encourages selective pressure for reversion of the deleterious mutation. Similar restoration of functional BRCA2 was identified in PEO1 cells following treatment with AG14361, a precursor of the PARPi talazoparib [40]. Accordingly, we propose that sub-dominant populations of OC cells harboring secondary reversion mutations undergo selection pressure against nonsense *BRCA2*^MUT^ in response to long-term treatment with olaparib. Post-treatment analysis of tumor biopsies revealed that most patients with platinum-resistant recurrent OC harboring secondary mutations restoring BRCA2 activity exhibited progressive disease following olaparib therapy [41]. Hence, awareness of the potential risk of developing resistance to olaparib in *BRCA2*^MUT^ tumors should be implemented in customized regimes involving combination treatment with ATR/CHK1 inhibitors to bypass PARPi limitations. Moreover, we demonstrated significant upregulation of full-length BRCA2 in PEO1-OR cells compared to sensitive parental cells, which could contribute to decreased sensitivity to olaparib. BRCA2 is one of the key factors promoting HR repair and is directly associated with diminished antitumor activity of olaparib [4,7]. We also confirmed the functionality of mutant BRCA2 in PEO1-OR cells in terms of RAD51 foci formation indicating proficiency in HR repair activity [15]. Recent studies have shown abrogated HR repair in a *BRCA2* missense variant harboring the R3052W mutation within the DNA binding domain due to disruption of translocation of BRCA2 to the nucleus and sequestering of RAD51 in the cytoplasm [74]. Using the RAD51 functional assay, we showed that PEO1-OR cells carrying the missense *BRCA2* variant Y1655L recruit RAD51 recombinase at DNA damage sites, whereby RAD51 co-localizes with a surrogate marker of DSBs, γH2AX. Similarly, PEO1 and PEO4 cells were able to induce RAD51 foci formation upon DNA damage. However, further work might be needed to comprehensively relate HRR functionality and activity with PARPi resistance in this model by HR reporter assay and/or by monitoring RAD51 foci formation in cells at the S/G2 phase where HR is most predominant.

While PARPi agents show the greatest efficacy in HRD tumors, efficacy has also been reported in a percentage of OC patients with HR proficiency [1,67]. Hence, we analyzed BRCA1 protein based on the finding that in HR-proficient tumors, RAD51 is recruited not only by BRCA2 but also by BRCA1 to promote HR repair [29,75]. BRCA1 is also an upstream regulator of BRCA2 that directly recruits BRCA2 at DSB sites via interactions with PALB2 [76]. Downregulation of *BRCA1* in OC cell lines has been shown to increase sensitivity to olaparib and decrease cell survival. Here, we demonstrated in PEO1-OR cells upregulation of basal levels of BRCA1, as well as in response to olaparib. Our findings support the hypothesis that decreased sensitivity to olaparib in OC cells is conferred through the induction of HR repair-promoting factors [77]. On the other hand, 53BP1 normally inhibits BRCA1 to favor NHEJ repair [16]. Notably, loss of *TP53BP1* in *BRCA1*^MUT^ OC cells was previously associated with increased HR activity as a mechanism of resistance to PARPi veliparib [5]. In our study, 53BP1 expression remained unchanged in BRCA1-proficient PEO1-OR cells, suggesting that accurate HR repair is favored over error-prone NHEJ through upregulation of BRCA1.

Notably, ATR/CHK1 signaling is associated with the regulation of HR repair. CHK1 kinase phosphorylates RAD51 and BRCA2 to facilitate RAD51-BRCA2 complex recruitment to DNA damage sites [78,79]. More recent studies have shown that BRCA1 phosphorylation at Thr1394 by ATR also plays an important role in promoting HR repair [80]. Our findings regarding BRCA1 and RAD51 upregulation in PEO1-OR in response to olaparib support the rationale for combining ATR/CHK1 pathway inhibitors with olaparib to disrupt HR repair and overcome resistance to PARPi. Accordingly, we addressed ATR/CHK1 axis activity, which orchestrates HR repair in the DDR pathway and may thus constitute a biomarker of PARPi response and a promising therapeutic target. The ATR/CHK1 pathway is also implicated in controlling G2/M checkpoint transition in *TP53*^MUT^ OC cells with abrogated S phase arrest to prevent entry into mitosis with damaged DNA [17,19,64,81]. However, the role of olaparib in the regulation of ATR/CHK1 is poorly understood in olaparib-resistant OC cells. Here, the ATR/CHK1 pathway was not activated in response to non-toxic doses of olaparib in PEO1-OR cells. Previous studies have also shown that treatment with PARPi alone has no impact on CHK1 phosphorylation in HGSOC cell lines with acquired and de novo resistance to olaparib [19]. Furthermore, contrary to PEO1-OR olaparib-resistant cells, PARPi-sensitive PEO1 HGSOC cells were arrested in the G2/M phase in response to olaparib, consistent with previous findings [19]. Cell cycle progression profiling with concurrent γH2AX monitoring highlighted that olaparib-resistant cells progressed normally through checkpoints with reduced DNA damage, indicative of genomic stability in the presence of PARPi. This observation confirms previous results showing that olaparib does not induce γH2AX accumulation in OC cells with intrinsic or acquired resistance to PARPi [19,82]. We propose that PEO1-OR cells limit their dependence on the ATR/CHK1 checkpoint pathway following treatment with olaparib at doses exerting significant cytotoxicity in sensitive OC cell lines.

Next, we addressed HR-independent modes of olaparib resistance as the absence of functional PARP1 itself, either due to loss of expression or inactivating mutations, may abrogate the cytotoxic mechanism of action of PARPi [83]. A PARP1 missense variant (p.V762A) was identified by us in PEO1 and PEO1-OR cells, which has been previously associated with decreased PARylation activity due to localization in the catalytic domain of PARP1 [84]. Our data are in keeping with previous findings showing that PARP1 expression remains unchanged in HGSOC cells with acquired resistance to olaparib [12] and no correlation between PARP1 levels and resistance to olaparib in clinical samples [5]. Noteworthily, in this study, treatment with DNA-damaging olaparib led to upregulation of PARP1 in olaparib-resistant cells, which may be correlated with PARP1’s ability to detect SSBs and DSBs mainly in late S and G2 cells [85]. Indeed, our cytogenetic and γH2AX analyses showed a significantly lower accumulation of structural chromosomal abnormalities and DSBs in PEO1-OR compared to olaparib-sensitive cells. These observations are in line with previous works showing that olaparib has minimal effect on DNA damage in *BRCA2*^MUT^ olaparib-resistant cells [68,82]. Regarding PARP signaling, loss of PARG is reported to contribute to decreased PARPi trapping and resistance. Moreover, loss or inhibition of PARG may increase cellular PARylation and decrease PARPi cytotoxicity [86], suggesting that endogenous PARG is needed for PARPi cytotoxicity. Further studies demonstrated that loss of PARG in HR-proficient HGSOC cells contributes to resistance to PARPi [12]. However, the same study also revealed that the generation of acquired resistance to olaparib from an HR-deficient OC cell line resulted in clones with unchanged or decreased levels of PARG [12]. Our results showed that basal expression of PARG in PEO1-OR remained unchanged compared to parental olaparib-sensitive cells downregulating PARG, suggesting that *BRCA2*^MUT^ OC cells do not acquire resistance to olaparib through loss of PARG. Overall, the observation of no changes in basal levels of PARP1 and PARG in PEO1-OR cell lines supports the theory that OC cells with restored BRCA2 protein activity confer resistance independently of the PARP signaling pathway.

Previous studies demonstrated that olaparib-resistant OC cell lines display upregulation of MDR1 and inhibition of this efflux pump, with non-specific MDR1 inhibitor veliparib [87,88] partially restoring sensitivity to PARPi [19]. However, verapamil is an L-type calcium channel blocker rather than a specific MDR1 inhibitor [89,90]. The MDR1 efflux pump was not upregulated in PEO1-OR cells in our study, indicating no contribution of increased drug efflux to olaparib resistance. In our biological model, MDR1 made no or minor contributions to acquired resistance to olaparib, based on results obtained with the specific third-generation inhibitor of MDR1 tariquidar [90], which is consistent with earlier reports that resistance to PARPi is acquired independently of the MDR1 efflux pump [91].

Furthermore, we explored the differences in response to olaparib treatment with concurrent blockage of the ATR/CHK1 pathway in HGSOC cells. The rationale for combination therapy is based on the concept that treatment with two or more agents with different molecular targets should induce a synergistic antitumor effect. Combinations of PARPi with inhibitors of the ATR/CHK1 pathway are under investigation for OC in vitro and in clinical trials for PARPi-naïve HGSOC patients and those who previously received PARPi [17,20,22,23,43,66,92]. However, further molecular studies are crucial to characterize the cellular response to this combination treatment in OC cells with distinct HR activities and resistance mechanisms. In OC cells, the majority of which are *TP53*^MUT^, the G1/S cell cycle checkpoint is lost, and tumor cells are dependent on the remaining checkpoints regulated by the ATR/CHK1 pathway. Hence, inhibitors of ATR or CHK1 can hinder cell cycle-dependent DNA repair and promote progression through the cell cycle with unrepaired DNA damage induced by olaparib. Moreover, our work has revealed genetic premises underlying the use of an inhibitor of ATR and CHK1 kinases in OC. One of the missense variants detected by WES in *ATR* (c.632T > C, p.M211T) in PEO1 and PEO1-OR cells is predicted to induce gain-of-function activity, supporting the idea behind the inhibition of ATR kinase. Interestingly, this uncharacterized alteration has been documented in numerous tumor and control samples from breast and/or ovarian cancer families [87]. We also showed that studied *BRCA2*^MUT^ HGSOC cell lines carry a nonsynonymous mutation in *CHEK1* (c.1411A > G, p.I471V) and we speculate that it has no impact on the binding of MK-8776 to CHK1, as it occurs outside the binding pocket [88]. Recent studies suggest that this *CHEK1* variant is extremely common in primary and recurrent HGSOC, but undetectable in a clear-cell carcinoma subtype of EOC [93]. Accordingly, we believe that ATR and CHK1 present favorable molecular targets for combination treatment of HGSOC.

In studies aimed at assessing the effectiveness of combined treatment, we selected the minimal doses of drugs that inhibited cell growth and proliferation more significantly in olaparib-sensitive (PEO1, PEO4) than in olaparib-resistant cells (PEO1-OR) in response to a relatively long 5-day treatment period, as effective PARP inhibition in vitro requires several rounds of cell division. Interestingly, PEO4 cells seemed to be more sensitive to olaparib after 5 days of incubation than PEO1 cells based on the results of the MTT assay, which was further confirmed by the colony formation capacity of olaparib-sensitive cells. However, previous reports showed that PEO1 and PEO4 cells may exert distinct sensitivity to PARPi [40,42], suggesting it depends on the type of inhibitor used. We believe that changes induced by olaparib are more dynamic in PEO1 than PEO4 cells (reduced metabolic activity, greater accumulation of DNA damage) and are revealed as soon as after two days due to the higher proliferation rate of PEO1 than PEO4 cells and olaparib’s cytotoxic mode of action. Here, we showed that olaparib combined with ATRi or CHK1i exerted early cellular changes occurring after 48 h including synergistic antitumor activity in terms of inhibiting cellular metabolic activity and colony formation capacity in olaparib-sensitive OC cell lines. Combinations of ATRi or CHK1i with PARPi were also effective in olaparib-resistant cells, most notably as a long-term effect of the strategy, confirmed based on synergistic reduction in colony formation capacity. Our results are in keeping with published data showing that olaparib combined with inhibitors of the ATR/CHK1 pathway act synergistically in BRCA1/2-proficient and BRCA1/2-deficient OC cells in vitro and in vivo [18,19,23]. Previous studies have shown that ATRi compounds can sensitize *BRCA1*-deficient OC cells with acquired resistance to olaparib. Given that PEO1-OR cells contain higher levels of BRCA1 compared to olaparib-sensitive cells, we speculate that the BRCA1 protein may partially contribute to desensitization to ATRi [91]. In agreement with data from cytotoxic assays, blockage of the ATR/CHK1 pathway enhanced the accumulation of olaparib-induced DSBs and chromosomal aberrations in olaparib-sensitive cells, whereas PEO1-OR cells were less susceptible. We propose that olaparib-induced mechanisms involved in upregulating HR repair-promoting factors contribute to the PEO1-OR response. Notably, truncated PARP1 was not detected in olaparib-resistant cells, in contrast to the significant increase in the parental cell line in response to the inhibitors and their combinations. As cleavage of PARP1 is a surrogate marker of apoptosis and truncated PARP1 directly induces this type of cell death [94], it is inferred that PEO1-OR cells are less susceptible to programmed cell death induced by drug combinations.

Levels of γH2AX, a surrogate marker of DSBs, were elevated following combination treatment, relative to each monotherapy in the olaparib-sensitive OC cell lines. Upon combination treatments, phosphorylation of H2AX was less significant in PEO1-OR than in olaparib-sensitive cells, in agreement with results from the comet assay and evaluation of chromosome aberrations. Our findings were confirmed by the assessment of γH2AX phosphorylation throughout the cell cycle. Modest genotoxic activity resulting from concurrent blockage of PARP1 and the ATR/CHK1 pathway had no impact on RAD51 expression in all OC cell lines. However, data from functional assays confirmed that the inhibitor compounds induced co-localization of RAD51 foci with γH2AX at DNA damage sites to promote the assembly of HR pathway repair machinery in OC cell lines. Moreover, olaparib and inhibitors of ATR/CHK1 synergistically suppressed NHEJ-mediating 53BP1 levels in PEO1-OR cells. Furthermore, blockage of the ATR/CHK1 pathway had no impact on olaparib-induced G2/M arrest in sensitive OC cell lines, which is in line with previous studies [25]. In contrast, a previous study by Brill et al. [81] showed that the addition of ATRi or CHK1i to PARPi released G2/M arrest to a limited extent in PEO1 and PEO4 cells. We speculate that following combination treatments, which induce modest DNA damage, PEO1-OR cells progress through the cell cycle with unrepaired DNA, which could contribute to partial resensitization to olaparib in the presence of inhibitors of the ATR or CHK1 pathway. Indeed, the ATR/CHK1 pathway was not activated in PEO1-OR cells in response to co-treatment with olaparib with ATRi, suggesting that cells were not arrested in the G2/M phase to repair DNA lesions. CHK1 and ATR are reported to promote HR repair via phosphorylation of repair factors, such as RAD51, BRCA1, and BRCA2 [78,79,80]. In PEO1-OR cells, combination treatment exerted modest but promising cytotoxic activity via upregulation of RAD51, BRCA1, and BRCA2 due to the inhibition of ATR and CHK1 kinases.

In this paper, we successfully identified mechanisms of resistance to PARPi in one *BRCA2*^MUT^ HGSOC cell line with decreased sensitivity to olaparib, which surely does not fully reflect the complexity of this phenomenon in heterogeneous OC cells. However, some olaparib-resistant OC cell lines have already been developed from OC cells harboring *BRCA1* or *BRCA2* mutations showing a 2.1-fold to 13-fold increase in resistance [18,19]. Nevertheless, our findings provide a valuable addition to the understanding of PARPi resistance, especially in terms of the resensitization of HGSOC cells to olaparib.

## 5. Conclusions

In this study, we showed that PARPi-resistant PEO1-OR cells exhibit decreased sensitivity to olaparib due to reduced susceptibility to PARPi-induced DNA damage and G2/M arrest. Mechanistically, PEO1-OR cells are homozygous for a *BRCA2* double mutation (c.[4964A > T; 4965C > G], p.Y1655L) and show upregulation of functional intact BRCA2, increased expression of HR-promoting BRCA1 with unchanged RAD51 and 53BP1 levels, partially restored PARP signaling, and decreased phosphorylation of H2AX. Resistance to PARPi was acquired irrespective of MDR1 efflux pump activity. Integration of diverse mechanisms of resistance resulted in reduced accumulation of DNA DSBs and structural chromosomal aberrations and allowed cells to progress normally through the ATR/CHK1-regulated cell cycle. At sublethal doses of inhibitors for olaparib-sensitive cells, combination treatments exerted promising synergistic antitumor effects on PEO1-OR cells. Moreover, co-treatment with ATR/CHK1 inhibitors and olaparib induced a synergistic decrease in cell viability and survival and increase in DNA damage in the cell models examined, most notably in olaparib-sensitive cells. These findings support the potential utility of combinations of inhibitors of the ATR/CHK1 pathway together with olaparib in avoiding or overcoming drug resistance of OC.

## Figures and Tables

**Table 1:** Whole-exome sequencing-based detection of high-quality exonic variants detected in *ABCB1*, *ATR*, *BRCA1*, *BRCA2*, *CHEK1*, *H2AX*, *PARP1*, and *TP53* genes in PEO1 and PEO1-OR cell lines.

Gene	Mutation	Gene Region	Amino Acid Change	Type of Mutation	Functional Impact	Computed Pathogenicity	CADD Score	Allele Fraction [% of Total Reads]		Maximal Population Allele Frequency
								PEO1	PEO1-OR	
*TP53*	c.731G > A	CDS	p.G244D	M	loss	pathogenic	27	100%	100%	0%
*BRCA1*	c.4837A > G	CDS	p.S1613G	M	normal	benign (!)	<10	100%	100%	49% (South Asian)
	c.2082C > T	CDS	p.S694S	S	normal	benign	<10	100%	100%	49% (South Asian)
	c.2311T > C	CDS	p.L771L	S	normal	benign	<10	100%	100%	49% (South Asian)
	c.2612C > T	CDS	p.P871L	M	normal	benign (!)	18	100%	100%	81% (African)
	c.3113A > G	CDS	p.E1038G	M	normal	benign (!)	14	100%	100%	49% (South Asian)
	c.3548A > G	CDS	p.K1183R	M	normal	benign (!)	<10	100%	100%	49% (South Asian)
	c.4308T > C	CDS	p.S1436S	S	normal	benign	<10	100%	100%	49% (South Asian)
	c.-1074C > G	5′ UTR	–	–	normal	benign	<10	100%	100%	82% (African)
	c.-134T > C	5′ UTR	–	–	normal	benign	<10	100%	100%	49% (South Asian)
*BRCA2*	c.4965C > G	CDS	p.Y1655 *	STOP	loss	pathogenic	33	100%	100%	0.008% (European)
	c.4964A > T	CDS	p.Y1655F	M	normal	uncertain	<10	34%	94%	0%
	c.3807T > C	CDS	p.V1269V	S	normal	benign	<10	100%	100%	19% (African)
	c.4563A > G	CDS	p.L1521L	S	normal	benign	<10	100%	100%	100% (Jewish)
	c.6513G > C	CDS	p.V2171V	S	normal	benign	<10	100%	100%	100% (Jewish)
	c.7397T > C	CDS	p.V2466A	M	normal	benign	<10	100%	100%	100% (Jewish)
	c.* 105A > C	3′ UTR	–	–	normal	benign	<10	100%	100%	23% (South Asian)
*PARP1*	c.2285T > C	CDS	p.V762A	M	loss	benign (!)	27	100%	100%	43% (East Asian)
	c.243C > T	CDS	p.D81D	S	normal	benign	12	100%	100%	43% (East Asian)
	c.852T > C	CDS	p.A284A	S	normal	benign	<10	100%	100%	81% (East Asian)
	c.-17G > C	5′ UTR	–	–	normal	benign	12	100%	100%	43% (East Asian)
*ATR*	c.632T > C	CDS	p.M211T	M	gain	benign (!)	14	32%	34%	73% (African)
	c.1776T > A	CDS	p.G592G	S	normal	benign	<10	31%	31%	79% (African)
	c.1815T > C	CDS	p.D605D	S	normal	benign (!)	<10	32%	31%	44% (Jewish)
	c.5208T > C	CDS	p.Y1736Y	S	normal	benign (!)	<10	29%	30%	45% (Jewish)
	c.7875G > A	CDS	p.Q2625Q	S	normal	benign	<10	32%	30%	97% (African)
*CHEK1*	c.1411A > G	CDS	p.I471V	M	gain	benign	14	100%	100%	99.98% (East Asian)
	c.* 28–3033C > G	3′ UTR	–	–	normal	benign	<10	53%	59% (LQ)	42% (South Asian)
*ABCB1*	c.210A > G	CDS	p.G70G	S	normal	benign	<10	100%	100%	100% (East Asian)
*H2AX*	c.-1420G > A	5′ UTR	–	–	normal	benign	<10	53%	35% (LQ)	64% (East Asian)

!—conflicting pathogenic criteria were computationally applied to a single variant (at least one pathogenic and one benign, as described in Section 3); 3′ UTR—3′ untranslated region; 5′ UTR—5′ untranslated region; CDS—protein-coding sequence; CADD score ranges from 1 to 99 (higher value responds to more deleterious cases, i.e., 10 indicates top 1% pathogenic variants, 20 indicates top 0.1% pathogenic variants); M—missense mutation; LQ—low-quality score for the variant allele call in one sample; S—synonymous mutation; STOP—stop-gain mutation; *—termiantion codon.

## Data Availability

The data presented in this study are available from the corresponding author upon a reasonable request.

## Supplementary Materials

The following supporting information can be downloaded at: https://www.mdpi.com/article/10.3390/cells12071038/s1, Figure S1: Growth curves and population doubling times for ovarian cancer (OC) cell lines; Figure S2: Qualitative Western blot analysis of full-length and truncated BRCA2 in PEO1, PEO1-OR, and PEO4 ovarian cancer (OC) cell lines; Figure S3: Effect of MDR1 inhibition with tariquidar or verapamil on cytotoxicity of the MDR1 substrate doxorubicin and olaparib, ATRi, CHK1i, or their combinations after 48 h of treatment in HepG2 cell line and qualitative Western blot analysis of MDR1 expression in untreated HepG2 cells; Figure S4: Quantitative Western blot analysis of PARP1 basal expression in untreated PEO1, PEO1-OR, and PEO4 ovarian cancer (OC) cell lines with a representative blot; Table S1: Key materials and reagents used in the study; Table S2: Primary antibodies used in the study. Table S3: Secondary antibodies used in the study; Table S4: High-quality exonic and intronic variants detected in *ABCB1*, *ATR*, *BRCA1*, *BRCA2*, *CHEK1*, *H2AX*, *PARP1*, *PARG*, *RAD51*, *TP53*, and *TP53BP1* genes in PEO1 and PEO1-OR cell lines by whole-exome sequencing.

## Abbreviations

A	ATR inhibitor (ceralasertib)
ABC	ATP-binding cassette
ATCC	American Type Culture Collection
ATR	ataxia telangiectasia and RAD3-related protein
ATRi	ATR inhibitor(s)
BRCA1	breast cancer type 1 susceptibility protein
BRCA2	breast cancer type 2 susceptibility protein
BrdU	bromodeoxyuridine
C	CHK1 inhibitor (MK-8776)
CDI	coefficient of drug interaction
CHK1	checkpoint kinase 1
CHK1i	CHK1 inhibitor(s)
DDR	DNA damage response
DSB	double-strand break
ECACC	The European Collection of Authenticated Cell Cultures
HGSOC	high-grade serous ovarian cancer
HI-FBS	heat-inactivated fetal bovine serum
IC_50_	concentration of drug required to reduce cell viability to 50%
MDR	multidrug resistance
MDR1	multidrug resistance protein 1
MDRi	multidrug resistance inhibitor(s)
MFI	geometric mean fluorescence intensity
NGS	next-generation sequencing
NHEJ	non-homologous end joining
O	olaparib
OC	ovarian cancer
ORF	open reading frame
PARG	poly(ADP-ribose) glycohydrolase
PARP1	poly(ADP-ribose) polymerase 1
PARPi	PARP inhibitor(s)
PDT	population doubling time
PEO1-OR	PEO1 olaparib-resistant cell line
QCI-IT	Qiagen Clinical Insight Interpret Translational
SF	surviving fraction
Tar	tariquidar
WES	whole-exome sequencing
