# Supplementary material for: Olaparib-Resistant BRCA2MUT Ovarian Cancer Cells with Restored BRCA2 Abrogate Olaparib-Induced DNA Damage and G2/M Arrest Controlled by the ATR/CHK1 Pathway for Survival
Source: Cells. 2023 Mar 29;12(7):1038. doi: 10.3390/cells12071038 (PMC10093185; doi:10.3390/cells12071038)
Supplement: Supplementary file 1 [file cells-12-01038-s001.zip › cells-2202160-supplementary.pdf]

Supplementary Information

Olaparib-Resistant *BRCA2*^{MUT} Ovarian Cancer Cells with Restored BRCA2 Abrogate Olaparib-Induced DNA Damage and G2/M Arrest Controlled by the ATR/CHK1 Pathway for Survival

Łukasz Biegała, Arkadiusz Gajek, Agnieszka Marczak, Aneta Rogalska *

Supplementary Figure S1 (Figure S1)

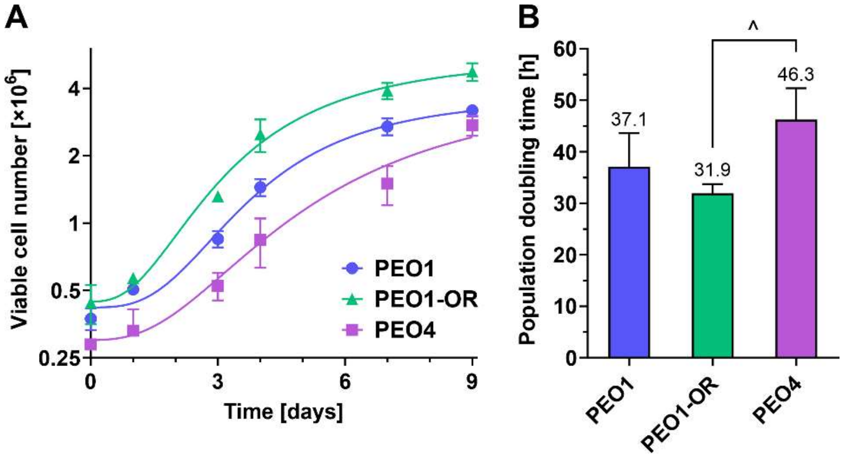

Figure S1. Growth curves and population doubling times for ovarian cancer (OC) cell lines. Cells seeded in 6-well plates were cultured up to 9 days and periodically subjected to endpoint counting from individual wells to determine the number of viable cells using trypan blue exclusion assay. (**A**) Growth curves of OC cell lines cultured under optimal conditions for 9 days. The results are shown as mean ± SD (n = 3). (**B**) Population doubling times of OC cell lines estimated in populations exhibiting exponential growth (days 2 – 5). Ordinary one-way ANOVA followed by Tukey's multiple comparison test was used to compare population doubling times between cell line: ^$p < 0.05$ statistically significant.

Supplementary Figure S2 (Figure S2)

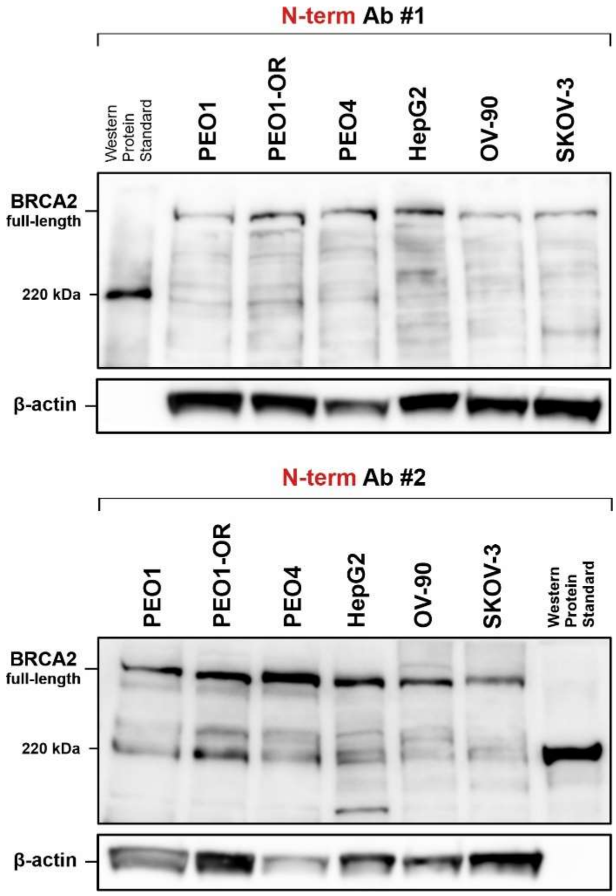

Figure S2. Qualitative western blot analysis of full-length and truncated BRCA2 in PEO1, PEO1-OR, and PEO4 ovarian cancer (OC) cell lines. HepG2, OV-90, and SKOV-3 cell lines were used as negative controls of truncated BRCA2 expression. Whole-cell lysates (30 µg of protein) were loaded into each lane and separated using 8% Bis-Tris gels by SDS-PAGE. Two various antibodies targeting different epitopes within N-terminus of the protein were used for immunodetection to confirm obtained results (N-term Ab #1 and #2). Sharp band from a western protein standard (MagicMark™ XP Western Protein Standard, Thermo Fisher Scientific) corresponds to the protein molecular weight of 220 kDa. Truncated form of BRCA2 (calculated molecular weigh of 186 kDa) was undetectable.

Supplementary Figure S3 (Figure S3)

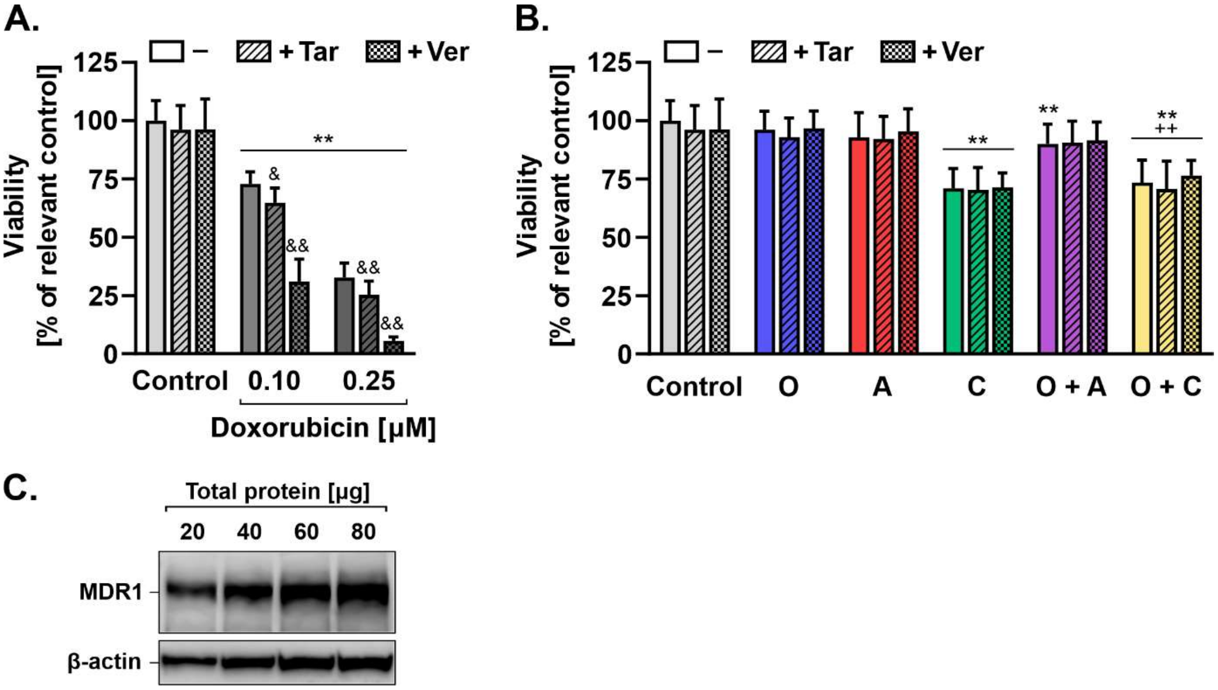

Figure S3. Effect of MDR1 inhibition with tariquidar (Tar) or verapamil (Ver) on cytotoxicity of a MDR1 substrate doxorubicin and olaparib, ATRi, CHK1i, or their combinations after 48 h of treatment in HepG2 cell line and qualitative western blot analysis of MDR1 expression in untreated HepG2 cells. Cell viability was assessed by MTT assay after treatment with **(A)** doxorubicin or **(B)** olaparib, ATRi, CHK1i, or their combinations, and expressed as the percentage of relevant control cells (untreated control for cells treated with tested drugs only; control with tariquidar or verapamil for cells treated with tested drugs and a relevant MDR1 inhibitor). Briefly, HepG2 cells were pre-treated with MDR inhibitors for 1 h (0.1 µM Tar or 50 µM Ver) following incubation with olaparib (O, 5 µM), ATRi (A, 0.5 µM), CHK1i (C, 2.5 µM) or their combinations in the absence and presence of MDR1 inhibitors. Co-treatment of tariquidar (0.1 µM, striped bars) or verapamil (50 µM, checked bars) with doxorubicin (0.10 µM and 0.25 µM) was used as a positive control of enhanced cytotoxicity of a known MDR1 substrate due to MDR1 inhibition. Data was expressed as mean ± SD (n = 3 – 4). Welch's ANOVA followed by Dunnett T3 multiple comparison test was used to compare viability of each group (with unequal variances) in response to the treatments: *$p < 0.05$, **$p < 0.01$ treatment vs. control without MDR1 inhibitor; &$p < 0.05$, &&$p < 0.01$ treatment vs. treatment with MDR1 inhibitor (tariquidar or verapamil). **(C)** Qualitative western blot analysis of MDR1 expression in untreated HepG2 cells confirming protein expression in the cells.

<u>**Supplementary Figure S4 (Figure S4)**</u>

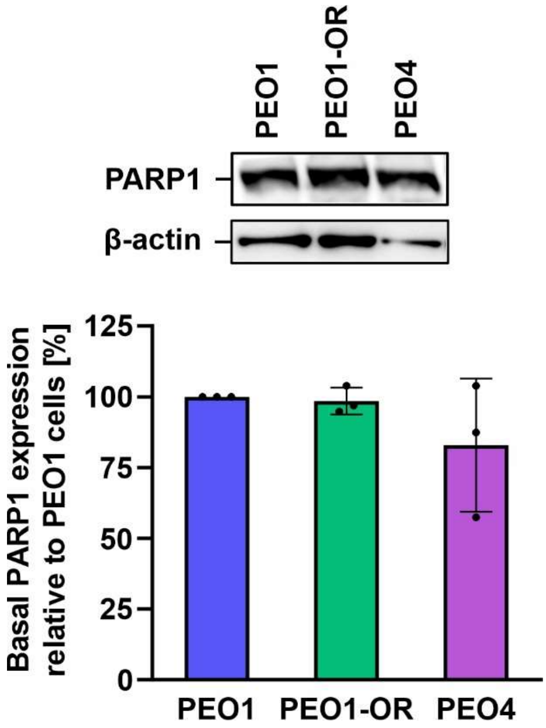

Figure S4. Quantitative western blot analysis of PARP1 basal expression in untreated PEO1, PEO1-OR, and PEO4 ovarian cancer (OC) cell lines with a representative blot. Whole-cell lysates (30 μg of protein) were loaded into each lane and separated in 8% Bis-Tris gels by SDS-PAGE following protein transfer and immunodetection. The results are shown as mean ± SD (n = 3). Individual data points are shown in bar charts presenting basal expression levels. Ordinary one-way ANOVA followed by Tukey's multiple comparison test was used to compare basal protein expression between cell lines: ^p < 0.05, ^^p < 0.01 differences between cell lines.

Supplementary Table S1 (Table S1)

Key materials and reagents used in the study.

Reagent	Catalogue Number	Manufacturer
(±)-Verapamil HCl	V4629	Sigma-Aldrich (Merk)
BD Pharmingen™ FITC BrdU Flow Kit	557891	BD Biosciences
Colcemid	10295892001	Roche (Merck)
Crystal violet	911517ZA	VWR
DMEM, high glucose (4.5 g/L), GlutaMAX™ Supplement, HEPES	32430-027	Gibco (Thermo Fisher Scientific)
Doxorubicin HCl	SRP04600d	Sequoia Research Products Limited
FBS, heat-inactivated, qualified	10270106	Gibco (Thermo Fisher Scientific)
GenElute™ Mammalian Genomic DNA Miniprep Kits	G1N10	Sigma-Aldrich (Merk)
Giemsa Dilution Buffer (20X)	1062.3	AQUA-MED
Giemsa Stain (10X)	1020.1	AQUA-MED
Halt™ Phosphatase Inhibitor Cocktail (100X)	78420	Thermo Fisher Scientific
Halt™ Protease Inhibitor Cocktail (100X)	87786	Thermo Fisher Scientific
HiMark™ Pre-stained Protein Standard	LC5699	Invitrogen (Thermo Fisher Scientific)
MagicMark™ XP Western Protein Standard		Invitrogen (Thermo Fisher Scientific)
MOPS Running Buffer	MPM0PS	Millipore (Merck)
mPAGE® 4X LDS Sample Buffer	MPSB	Millipore (Merck)
mPAGE® Bis-Tris Precast Gels 10%	MP10W10	Millipore (Merck)
mPAGE® Bis-Tris Precast Gels 12%	MP12W10	Millipore (Merck)
mPAGE® Bis-Tris Precast Gels 8%	MP8W10	Millipore (Merck)
mPAGE® Transfer Buffer	MPTRB	Millipore (Merck)
MTT	20395.03	SERVA Electrophoresis
Pierce™ Detergent Compatible Bradford	23246	Thermo Fisher Scientific
PVDF Membrane	IPVH85R	Millipore (Merck)
RIPA Lysis and Extraction Buffer	89900	Thermo Fisher Scientific
RPMI 1640, GlutaMAX™ Supplement, HEPES	72400-021	Gibco (Thermo Fisher Scientific)
Spectra™ Multicolor Broad Range Protein Ladder	26634	Thermo Fisher Scientific
Spectra™ Multicolor High Range Protein Ladder	26625	Thermo Fisher Scientific
SuperBlock™ (TBS) Blocking Buffer	37581	Thermo Fisher Scientific
SuperSignal™ West Atto Ultimate Sensitivity Substrate	A38555	Thermo Fisher Scientific

SuperSignal™ West Pico PLUS Chemiluminescent Substrate	34577	Thermo Fisher Scientific
Tariquidar	SML1790	Sigma-Aldrich (Merk)
Trypsin-EDTA	25200072	Gibco (Thermo Fisher Scientific)
β-mercaptoethanol	M6250	Sigma-Aldrich (Merck)

Supplementary Table S2 (Table S2)

Primary antibodies used in the study.

Target	Host and Clonality	Supplier	Catalogue Number	Dilution	Dilution buffer	Blocking agent	Application
53BP1	Mouse monoclonal	Sigma-Aldrich (Merck)	MAB3802	1:1000	5% BSA in TBST	SuperBlock™ (TBS) Blocking Buffer	WB
ATR	Mouse monoclonal	Invitrogen (Thermo Fisher Scientific)	MA1-23158	1:1000	5% BSA in TBST	5% non-fat milk in TBST	WB
BRCA1	Rabbit polyclonal	Cell Signaling Technology	9010	1:1000	5% BSA in TBST	SuperBlock™ (TBS) Blocking Buffer	WB
BRCA2 (N-term) #1	Rabbit polyclonal	Thermo Fisher Scientific	A303-434A	1:10 000	5% non-fat milk in TBST	SuperBlock™ (TBS) Blocking Buffer	WB
BRCA2 (N-term) #2	Mouse monoclonal	Invitrogen (Thermo Fisher Scientific)	MA523942	1:1000	5% non-fat milk in TBST	SuperBlock™ (TBS) Blocking Buffer	WB
BRCA2 (C-term)	Rabbit polyclonal	Thermo Fisher Scientific	A300-005A	1:1000	5% BSA in TBST	SuperBlock™ (TBS) Blocking Buffer	WB
CHK1	Rabbit monoclonal	Thermo Fisher Scientific	MA5-32180	1:1000	5% BSA in TBST	5% non-fat milk in TBST	WB
MDR1	Rabbit monoclonal	Invitrogen (Thermo Fisher Scientific)	MA5-32282	1:1000	5% BSA in TBST	SuperBlock™ (TBS) Blocking Buffer	WB
PARG	Mouse monoclonal	Invitrogen (Thermo Fisher Scientific)	MA5-27034	1:1000	5% BSA in TBST	SuperBlock™ (TBS) Blocking Buffer	WB

Target	Host and Clonality	Supplier	Catalogue Number	Dilution	Dilution buffer	Blocking agent	Application
PARP1	Rabbit monoclonal	Cell Signaling Technology	9532	1:1000	5% BSA in TBST	5% non-fat milk in TBST	WB
pATR (Thr1989)	Rabbit monoclonal	Cell Signaling Technology	30632	1:1000	5% BSA in TBST	5% non-fat milk in TBST	WB
pCHK1 (Ser345)	Rabbit monoclonal	Cell Signaling Technology	2348	1:1000	5% BSA in TBST	5% non-fat milk in TBST	WB
RAD51	Rabbit polyclonal	Sigma-Aldrich (Merck)	ABE257	1:1000 (WB)<br>1:500 (IF)	5% BSA in TBST	SuperBlock™ (TBS) Blocking Buffer	WB, IF
β-actin	Mouse monoclonal	Sigma-Aldrich (Merck)	A1978	1:10 000	5% BSA in TBST	5% non-fat milk in TBST or SuperBlock™ (TBS) Blocking Buffer	WB
γH2AX (Ser139)	Mouse monoclonal	Sigma-Aldrich (Merck)	05-636	1:1000 (WB)<br>1:500 (IF)	5% BSA in TBST	SuperBlock™ (TBS) Blocking Buffer	WB, IF
γH2AX (Ser139)	Mouse monoclonal	Invitrogen (Thermo Fisher Scientific)	12-9865-42	1:20	N/A	1X BD Perm/Wash Buffer	FC

Supplementary Table S3 (Table S3)

Secondary antibodies used in the study.

Species Reactivity	Conjugate	Host	Manufacturer	Catalogue Number	Dilution	Dilution buffer	Application
Mouse	HRP	Goat	Invitrogen (Thermo Fisher Scientific)	A28177	1:10 000	5% non-fat milk in TBST	WB
Rabbit	HRP	Goat	Cell Signaling Technology	7074	1:10 000		WB
Mouse	Alexa Fluor™ Plus 488	Goat	Invitrogen (Thermo Fisher Scientific)	A32723	1:1000	DPBS 1X with 1% BSA and 0.3% Triton X-100	IF
Rabbit	Alexa Fluor® 555	Goat	Cell Signaling Technology	4413	1:1000		IF

Supplementary Table S4 (Table S4)

High-quality exonic and intronic variants detected in *ABCB1*, *ATR*, *BRCA1*, *BRCA2*, *CHEK1*, *H2AX*, *PARP1*, *PARG*, *RAD51*, *TP53*, and *TP53BP1* genes in PEO1 and PEO1-OR cell lines by whole-exome sequencing.

Gene	Mutation	Gene region	Variant type	Amino acid change	Type of mutation	Functional impact	Computed pathogenicity	CADD score	Allele fraction [% of total reads]		Maximal population allele frequency
									PEO1	PEO1-OR	
TP53	c.731G>A	CDS	SNV	p.G244D	missense	loss	pathogenic	27	**100%** (of 83 reads)	**100%** (of 90 reads)	0%
	c.993+409delT	intron	deletion	–	–	normal	benign	<10	**53%** (of 19 reads)	**absent**	11% (African)
BRCA1	c.4837A>G	CDS	SNV	p.S1613G	missense	normal	benign **(!)**	<10	**100%** (of 140 reads)	**100%** (of 180 reads)	49% (South Asian)
	c.2082C>T	CDS	SNV	p.S694S	synonymous	normal	benign	<10	**100%** (of 183 reads)	**100%** (of 242 reads)	49% (South Asian)
	c.2311T>C	CDS	SNV	p.L771L	synonymous	normal	benign	<10	**100%** (of 197 reads)	**100%** (of 262 reads)	49% (South Asian)
	c.2612C>T	CDS	SNV	p.P871L	missense	normal	benign **(!)**	18	**100%** (of 135 reads)	**100%** (of 159 reads)	81% (African)
	c.3113A>G	CDS	SNV	p.E1038G	missense	normal	benign **(!)**	14	**100%** (of 189 reads)	**100%** (of 240 reads)	49% (South Asian)
	c.3548A>G	CDS	SNV	p.K1183R	missense	normal	benign **(!)**	<10	**100%** (of 190 reads)	**100%** (of 198 reads)	49% (South Asian)
	c.4308T>C	CDS	SNV	p.S1436S	synonymous	normal	benign	<10	**100%** (of 120 reads)	**100%** (of 160 reads)	49% (South Asian)
	c.-1074C>G	5' UTR	SNV	–	–	normal	benign	<10	**100%** (of 70 reads)	**100%** (of 109 reads)	82% (African)
	c.-134T>C	5' UTR	SNV	–	–	normal	benign	<10	**100%** (of 32 reads)	**100%** (of 32 reads)	49% (South Asian)
	c.5468-121delA	intron	deletion	–	–	normal	uncertain significance	<10	**50%** (of 20 reads)	**absent**	0.40% (East Asian)
	c.4987-144_4987-142dupAAA	intron	insertion	–	–	normal	likely benign	<10	**absent**	**100%** (of 23 reads)	4% (African)
	c.134+224delT	intron	deletion	–	–	normal	benign	<10	**94%** (of 16 reads)	**95%** (of 20 reads)	52% (South Asian)
	c.213-161A>G	intron	SNV	–	–	normal	benign	<10	**100%** (of 37 reads)	**100%** (of 40 reads)	82% (African)
	c.441+36_441+49 delCTTTTCTTTTTTTT	intron	deletion	–	–	normal	benign	<10	**100%** (of 68 reads)	**97%** (of 131 reads)	49% (South Asian)

Gene	Mutation	Gene region	Variant type	Amino acid change	Type of mutation	Functional impact	Computed pathogenicity	CADD score	Allele fraction [% of total reads]		Maximal population allele frequency
									PEO1	PEO1-OR	
	c.441+64delT	intron	deletion	–	–	normal	benign	<10	**100%** (of 65 reads)	**94%** (of 126 reads)	75% (African)
	c.547+146A>T	intron	SNV	–	–	normal	benign	<17	**100%** (of 25 reads)	**100%** (of 25 reads)	49% (South Asian)
	c.548-58delT	intron	deletion	–	–	normal	benign	<12	**100%** (of 93 reads)	**100%** (of 89 reads)	49% (South Asian)
	c.593+167T>C	intron	SNV	–	–	normal	benign	<10	**100%** (of 34 reads)	**100%** (of 49 reads)	49% (South Asian)
	c.4097-141A>C	intron	SNV	–	–	normal	benign	<10	**100%** (of 41 reads)	**100%** (of 67 reads)	67% (African)
	c.4357+117G>A	intron	SNV	–	–	normal	benign	<10	**100%** (of 19 reads)	**100%** (of 45 reads)	12% (South Asian)
	c.4358-2885G>A	intron	SNV	–	–	normal	benign	<10	**100%** (of 109 reads)	**100%** (of 150 reads)	49% (South Asian)
	c.4358-2590T>G	intron	SNV	–	–	normal	benign	<10	**100%** (of 18 reads)	**100% (LQ)** (of 24 reads)	49% (South Asian)
	c.4485-203_4485-199 delAACCC	intron	deletion	–	–	normal	benign	<10	**100%** (of 12 reads)	**100%** (of 7 reads)	49% (South Asian)
	c.4485-137T>A	intron	SNV	–	–	normal	benign	<10	**100%** (of 29 reads)	**100%** (of 23 reads)	69% (African)
	c.4485-63C>G	intron	SNV	–	–	normal	benign	<10	**100%** (of 62 reads)	**100%** (of 63 reads)	49% (South Asian)
	c.4987-92A>G	intron	SNV	–	–	normal	benign	<10	**100%** (of 47 reads)	**100%** (of 68 reads)	49% (South Asian)
	c.4987-68A>G	intron	SNV	–	–	normal	benign **(!)**	<10	**100%** (of 67 reads)	**100%** (of 104 reads)	49% (South Asian)
	c.5074+65G>A	intron	SNV	–	–	normal	benign	<10	**100%** (of 85 reads)	**100%** (of 138 reads)	46% (South Asian)
	c.5152+66G>A	intron	SNV	–	–	normal	benign	11	**100%** (of 74 reads)	**100%** (of 85 reads)	49% (South Asian)
BRCA2	c.4965C>G	CDS	SNV	p.Y1655*	stop gain	loss	pathogenic	33	**100%** (of 64 reads)	**100%** (of 78 reads)	0.008% (European)
	c.4964A>T	CDS	SNV	p.Y1655F	missense	normal	uncertain significance	<10	**34%** (of 64 reads)	**94%** (of 79 reads)	0%
	c.3807T>C	CDS	SNV	p.V1269V	synonymous	normal	benign	<10	**100%** (of 56 reads)	**100%** (of 81 reads)	19% (African)
	c.4563A>G	CDS	SNV	p.L1521L	synonymous	normal	benign	<10	**100%** (of 77 reads)	**100%** (of 105 reads)	100% (Jewish)

Gene	Mutation	Gene region	Variant type	Amino acid change	Type of mutation	Functional impact	Computed pathogenicity	CADD score	Allele fraction [% of total reads]		Maximal population allele frequency
									PEO1	PEO1-OR	
	c.6513G>C	CDS	SNV	p.V2171V	synonymous	normal	benign	<10	**100%** (of 88 reads)	**100%** (of 84 reads)	100% (Jewish)
	c.7397T>C	CDS	SNV	p.V2466A	missense	normal	benign	<10	**100%** (of 67 reads)	**100%** (of 83 reads)	100% (Jewish)
	c.*105A>C	3′ UTR	SNV	–	–	normal	benign	<10	**100%** (of 30 reads)	**100%** (of 37 reads)	23% (South Asian)
	c.793+98G>A	intron	SNV	–	–	normal	benign	<10	**100%** (of 49 reads)	**100%** (of 48 reads)	100% (Jewish)
	c.6938-120T>C	intron	SNV	–	–	normal	benign	<10	**100% (LQ)** (of 16 reads)	**100%** (of 21 reads)	100% (Jewish)
PARP1	c.2285T>C	CDS	SNV	p.V762A	missense	loss	benign **(!)**	27	**100%** (of 49 reads)	**100%** (of 36 reads)	43% (East Asian)
	c.243C>T	CDS	SNV	p.D81D	synonymous	normal	benign	12	**100%** (of 106 reads)	**100%** (of 127 reads)	43% (East Asian)
	c.852T>C	CDS	SNV	p.A284A	synonymous	normal	benign	<10	**100%** (of 56 reads)	**100%** (of 52 reads)	81% (East Asian)
	c.-17G>C	5' UTR	SNV	–	–	normal	benign	12	**100%** (of 32 reads)	**100%** (of 71 reads)	43% (East Asian)
	c.286+206G>A	intron	SNV	–	–	normal	benign	<10	**100%** (of 45 reads)	**100%** (of 83 reads)	81% (East Asian)
	c.617+64C>A	intron	SNV	–	–	normal	benign	<10	**100%** (of 60 reads)	**100%** (of 114 reads)	43% (East Asian)
	c.617+12A>G	intron	SNV	–	–	normal	benign	<10	**100%** (of 83 reads)	**100%** (of 43 reads)	94% (East Asian)
	c.717+87T>C	intron	SNV	–	–	normal	benign	<10	**100%** (of 47 reads)	**100%** (of 106 reads)	47% (East Asian)
ATR	c.632T>C	CDS	SNV	p.M211T	missense	gain	benign **(!)**	14	**32%** (of 285 reads)	**34%** (of 359 reads)	73% (African)
	c.1776T>A	CDS	SNV	p.G592G	synonymous	normal	benign	<10	**31%** (of 1002 reads)	**31%** (of 1133 reads)	79% (African)
	c.1815T>C	CDS	SNV	p.D605D	synonymous	normal	benign **(!)**	<10	**32%** (of 1108 reads)	**31%** (of 1274 reads)	44% (Jewish)
	c.5208T>C	CDS	SNV	p.Y1736Y	synonymous	normal	benign **(!)**	<10	**29%** (of 172 reads)	**30%** (of 185 reads)	45% (Jewish)
	c.7875G>A	CDS	SNV	p.Q2625Q	synonymous	normal	benign	<10	**32%** (of 335 reads)	**30%** (of 345 reads)	97% (African)

Gene	Mutation	Gene region	Variant type	Amino acid change	Type of mutation	Functional impact	Computed pathogenicity	CADD score	Allele fraction [% of total reads]		Maximal population allele frequency
									PEO1	PEO1-OR	
	c.1885+97_1885+100 delATTT	intron	deletion	–	–	normal	benign	<10	**32%** (of 462 reads)	**32%** (of 532 reads)	44% (Jewish)
	c.2341+185G>A	intron	SNV	–	–	normal	benign	<10	**43%** (of 444 reads)	**39%** (of 322 reads)	48% (African)
	c.3357+128G>A	intron	SNV	–	–	normal	benign	<10	**38%** (of 48 reads)	**34%** (of 53 reads)	24% (Jewish)
	c.4852+39delT	intron	deletion	–	–	normal	uncertain significance	<10	**41%** (of 87 reads)	**absent**	0.3% (South Asian)
	c.5288+130G>A	intron	SNV	–	–	normal	benign	<10	**31%** (of 62 reads)	**29%** (of 75 reads)	45% (Jewish)
	c.5898+25T>G	intron	SNV	–	–	normal	benign	<10	**75%** (of 32 reads)	**85%** (of 34 reads)	79% (African)
	c.5898+82delA	intron	deletion	–	–	normal	benign	<10	**100%** (of 19 reads)	**100%** (of 22 reads)	71% (African)
	c.5898+67_5898+79 delTATATATATATAT	intron	deletion	–	–	normal	benign	<10	**100%** (of 19 reads)	**59%** (of 17 reads)	39% (East Asian)
	c.5898+65_5898+79 delTATATATATATATA T	intron	deletion	–	–	normal	benign	<10	**absent**	**41%** (of 17 reads)	5% (Jewish)
	c.5898+54A>G	intron	SNV	–	–	normal	benign	<10	**100%** (of 18 reads)	**62% (LQ)** (of 21 reads)	30% (African)
	c.5898+52A>G	intron	SNV	–	–	normal	likely benign	<10	**absent**	**24%** (of 21 reads)	3% (Jewish)
	c.1171-58A>C	intron	SNV	–	–	normal	benign	10	**64%** (of 70 reads)	**67%** (of 101 reads)	6% (European)
	c.2342-175A>G	intron	SNV	–	–	normal	benign	<10	**33%** (of 18 reads)	**28% (LQ)** (of 29 reads)	44% (Jewish)
	c.2634-37_2634-36dupGT	intron	insertion	–	–	normal	benign	<10	**89%** (of 142 reads)	**86%** (of 170 reads)	42% (European)
	c.2634-74C>T	intron	SNV	–	–	normal	benign	<10	**34%** (of 156 reads)	**34%** (of 217 reads)	79% (African)
	c.1411A>G	CDS	SNV	p.I471V	missense	gain	benign	14	**100%** (of 105 reads)	**100%** (of 105 reads)	99.98% (East Asian)
CHEK1	c.*28-3033C>G	3′ UTR	SNV	–	–	normal	benign	<10	**53%** (of 17 reads)	**59% (LQ)** (of 22 reads)	42% (South Asian)
	c.66-36G>T	intron	SNV	–	–	normal	benign	<10	**52%** (of 79 reads)	**57%** (of 94 reads)	42% (East Asian)

Gene	Mutation	Gene region	Variant type	Amino acid change	Type of mutation	Functional impact	Computed pathogenicity	CADD score	Allele fraction [% of total reads]		Maximal population allele frequency
									PEO1	PEO1-OR	
	c.1101+111T>A	intron	SNV	–	–	normal	benign	<10	**45%** (of 91 reads)	**51%** (of 92 reads)	42% (East Asian)
	c.1233+35G>A	intron	SNV	–	–	normal	benign	<10	**52%** (of 126 reads)	**42%** (of 132 reads)	57% (Latino)
ABCB1	c.210A>G	CDS	SNV	p.G70G	synonymous	normal	benign	<10	**100%** (of 109 reads)	**100%** (of 100 reads)	100% (East Asian)
	c.530+139C>T	intron	SNV	–	–	normal	benign	<10	**100%** (of 24 reads)	**100%** (of 39 reads)	64% (East Asian)
RAD51	c.645-108delA	intron	deletion	–	–	normal	uncertain significance	–	**absent**	**37%** (of 57 reads)	0%
	c.645-110_645-108delAAA	intron	deletion	–	–	normal	likely benign	<10	**absent**	**54%** (of 57 reads)	2.8% (African)
	c.225+190delT	intron	deletion	–	–	normal	benign	<10	**86%** (of 69 reads)	**100%** (of 400 reads)	64% (East Asian)
	c.226-70T>A	intron	SNV	–	–	normal	benign	<10	**68%** (of 79 reads)	**55%** (of 89 reads)	36% (European)
	c.226-33T>G	intron	SNV	–	–	normal	benign	11	**26%** (of 109 reads)	**38%** (of 118 reads)	67% (East Asian)
H2AX	c.-1420G>A	5′ UTR	SNV	–	–	normal	benign	<10	**53%** (of 19 reads)	**35% (LQ)** (of 34 reads)	64% (East Asian)
TP53BP1	c.3813+58delT	intron	deletion	–	–	normal	uncertain significance	<10	**28%** (of 347 reads)	**26%** (of 415 reads)	0.48% (Latino)
	c.1165+217delT	intron	deletion	–	–	normal	likely benign	<10	**absent**	**64%** (of 11 reads)	1.6% (South Asian)
	c.272-49G>A	intron	SNV	–	–	normal	benign	<10	**100%** (of 165 reads)	**100%** (of 185 reads)	100% (Jewish)
PARG	c.1456-88_1456-87dupTA	intron	insertion	–	–	normal	benign	<10	**100%** (of 45 reads)	**100%** (of 70 reads)	59% (East Asian)
	c.1663-116A>C	intron	SNV	–	–	normal	benign	<10	**100%** (of 58 reads)	**100%** (of 84 reads)	59% (East Asian)
	c.1830+132_1830+133 delAG	intron	deletion	–	–	normal	benign	<10	**20%** (of 35 reads)	**17%** (of 23 reads)	32% (European)

Color scale visualizes allele frequency of each variants (red – absent or low, yellow – moderate, green – high); **!** – conflicting pathogenic criteria has been computationally-applied to a single variant (at least one pathogenic and one benign criterion described in Results section); **3′ UTR** – 3′ untranslated region; **5′ UTR** – 5′ untranslated region; **CDS** – protein coding sequence; **CADD score** ranges from 1 to 99 (the higher the value the more deleterious case i.e., 10 indicates top 1% pathogenic variants, 20 indicates top 0.1% pathogenic variants); **LQ** – low quality score for the variant allele call in one sample. **Maximal population allele frequency** was obtained from The Genome Aggregation Database (gnomAD).
